# Photoperiod Extension Enhances Sexual Megaspore Formation and Triggers Metabolic Reprogramming in Facultative Apomictic *Ranunculus auricomus*

**DOI:** 10.3389/fpls.2016.00278

**Published:** 2016-03-08

**Authors:** Simone Klatt, Franz Hadacek, Ladislav Hodač, Gina Brinkmann, Marius Eilerts, Diego Hojsgaard, Elvira Hörandl

**Affiliations:** ^1^Albrecht-von-Haller-Institute for Plant Sciences, Department of Systematics, Biodiversity and Evolution of Plants (with Herbarium), Georg-August-University of Göttingen, Göttingen, Germany; ^2^Albrecht-von-Haller-Institute for Plant Sciences, Department of Plant Biochemistry, Georg-August-University of Göttingen, Göttingen, Germany

**Keywords:** apomixis, FCSS, light stress, oxidative stress, *Ranunculus*, reproduction mode, secondary metabolites, seed formation

## Abstract

Meiosis, the key step of sexual reproduction, persists in facultative apomictic plants functional to some extent. However, it still remains unclear how and why proportions of reproductive pathways vary under different environmental stress conditions. We hypothesized that oxidative stress mediates alterations of developmental pathways. In apomictic plants we expected that megasporogenesis, the stage directly after meiosis, would be more affected than later stages of seed development. To simulate moderate stress conditions we subjected clone-mates of facultative apomictic *Ranunculus auricomus* to 10 h photoperiods, reflecting natural conditions, and extended ones (16.5 h). Reproduction mode was screened directly after megasporogenesis (microscope) and at seed stage (flow cytometric seed screening). Targeted metabolite profiles were performed with HPLC–DAD to explore if and which metabolic reprogramming was caused by the extended photoperiod. Prolonged photoperiods resulted in increased frequencies of sexual vs. aposporous initials directly after meiosis, but did not affect frequencies of sexual vs. asexual seed formation. Changes in secondary metabolite profiles under extended photoperiods affected all classes of compounds, and c. 20% of these changes separated the two treatments. Unexpectedly, the renowned antioxidant phenylpropanoids and flavonoids added more to clone-mate variation than to treatment differentiation. Among others, chlorophyll degradation products, non-assigned phenolic compounds and more lipophilic metabolites also contributed to the dissimilarity of the metabolic profiles of plants that had been exposed to the two different photoperiods. The hypothesis of moderate light stress effects was supported by increased proportions of sexual megaspore development at the expense of aposporous initial formation. The lack of effects at the seed stage confirms the basic assumption that only meiosis and sporogenesis would be sensitive to light stress. The concomitant change of secondary metabolite profiles, as a systemic response at this early developmental stage, supports the notion that oxidative stress could have affected megasporogenesis by causing the observed metabolic reprogramming. Hypotheses of genotype-specific responses to prolonged photoperiods are rejected.

## Introduction

Sexual reproduction is common in eukaryotes compared to asexual reproduction, notwithstanding relatively high costs. Meiosis, the key step of eukaryotic sex, can break up favorable gene combinations; furthermore, sexual reproduction requires a mating partner (Maynard Smith, 1978; Bell, 1982; Otto, 2009). As yet, the benefits of sex are still not understood well. About 20 different hypotheses exist that attempt explaining the phenomenon (Birdsell and Wills, 2003). One often voiced advantage for asexual reproduction is that favorable gene combinations can be transferred faithfully to the offspring (Otto, 2009). Apomixis in plants, the asexual reproduction via seed, has evolved in many angiosperm lineages and occurs in nearly 300 genera (http://www.apomixis.uni-goettingen.de, Hojsgaard et al., 2014b). Gametophytic apomixis reduces reproductive costs, because it combines the development of an embryo sac out of an unreduced cell of the ovule (apomeiosis) with the parthenogenetic development of the egg cell without fertilization (Asker and Jerling, 1992). Nevertheless, also in apomictic plants sexual reproduction can occur within the same generation, which means that the reproduction mode is facultative (Aliyu et al., 2010; Cosendai et al., 2013; Dobeš et al., 2013; Hojsgaard et al., 2013, 2014a).

It is still unclear which factors influence the frequency of sex in the facultative apomictic plants. In terms of a long-term effect, sexual production might help to eliminate deleterious mutations (Muller, 1964; Hörandl, 2009; Hojsgaard and Hörandl, 2015), but robust hypotheses for a short-term benefit of sex are lacking still. The influence of various abiotic factors on flowering, such as temperature, length of the photoperiod, and drought stress, has been reported repeatedly (Keller and Körner, 2003; Lokhande et al., 2003; Sakai et al., 2006; Kurepin et al., 2007; Amasino, 2010). Experimental studies in several grass species provided evidence that a prolonged photoperiod leads to an increase in sexual embryo sac frequencies compared to apomeiotic ones (Evans and Knox, 1969: *Themeda*; Gupta et al., 1969; Saran and Dewet, 1976: *Dichantium*; Quarin, 1986: *Paspalum*). These results concur with observations that different kinds of environmental stress can increase the proportion of sexual offspring in various asexual/sexual eukaryotes (Bernstein, 1998; Nedelcu et al., 2004; Šarhanová et al., 2012; Speijer et al., 2015).

The physiological background of these phenomena, however, is still unclear. In plants, asexual reproduction is heritable and under control of the same genes that regulate the sexual pathway. The reproductive phenotype is mostly determined by temporal or spatial de-regulation of genes acting during female reproduction (Koltunow and Grossniklaus, 2003; Grimanelli, 2012; Galla et al., 2015). Variation in meiotic vs. apomeiotic embryo sac formation was suggested to be caused by dosage-effects of apomeiosis-controlling alleles and hence seems to be genotype-specific (Ozias-Akins and Van Dijk, 2007). The second step of reproduction, i.e., fertilization vs. parthenogenetic development of the egg cell, is under independent genetic control (Ozias-Akins and Van Dijk, 2007; Grimanelli, 2012). In facultative apomicts, proportions of functional megaspores, meiotically formed embryo sacs and finally sexually formed seeds can differ significantly within the same clone, implicating that also other processes influence gametogenesis and seed formation (Hojsgaard et al., 2013, 2014a; Hojsgaard and Hörandl, 2015).

A causal explanation for stress-dependent variation of reproduction modes, and for stage-specific stress-sensitivity, is still missing. So far, expression studies of female gametophytes of apomictic *Boechera* species, a close relative to *Arabidopsis*, revealed changes in the expression patterns of several hundred genes when sexual development switches to apomixis. The majority of transcripts comprised metabolism and developmental genes besides transcription factors with strongly stage-specific expression profiles (Sharbel et al., 2010; Schmidt et al., 2014). The Schmidt et al. (2014) study specifically points out that genes that are involved in the biosynthesis of spermidine and other polyamines are more expressed in the apomictic gametophyte. Polyamines can affect gene expression, cell proliferation, modulation of cell signaling, the permeability of certain ion channels and membrane stabilization. Polyamines are not only essential for growth but are involved in responses to stress and various diseases (Kusano et al., 2008). Accordingly, Schmidt et al. (2014) argue that this enhanced accumulation of polyamine might result in better protection against oxidative stress. In flower buds of *Arabidopsis* and many other plants, the predominant identified secondary (specialized) metabolites are phenylpropanoid polyamine conjugates (Fellenberg et al., 2009). Facultative sexual reproduction in apomictic angiosperms might be affected by oxidative stress in the ovule. An extended photoperiod can cause photo-oxidative stress (Foyer et al., 1994). The oxidative stress initiation hypothesis (Hörandl and Hadacek, 2013) posits that oxidative stress could activate meiosis-specific proteins that initiate double strand break formation and thus increase recombination frequency. Presently and before, suggestions have been voiced that meiotic sex might represent a cellular survival strategy (Bernstein et al., 1988; Bernstein, 1998; Hörandl and Hadacek, 2013; Speijer et al., 2015). This hypothesis influenced the design of this study decisively. Just like all other aerobic organisms, plants depend on mechanisms that contribute to mitigating of the potential damage caused by the enzymatic and non-enzymatic overproduction of highly reactive oxygen species (ROS) by various forms of abiotic and biotic stress in attempts to protect the integrity of essential organelles, mitochondria, chloroplasts and peroxisomes representing those most endangered. ROS, such as superoxide anion, O_2_^•-^, hydroxyl radical, ^•^OH, hydrogen peroxide, H_2_O_2_, and singlet oxygen, ^1^O_2_, have to be considered not only as pure toxic cellular stress by-products but as important signaling components that can regulate various metabolic activities (Fujita et al., 2006; Suzuki et al., 2012).

Following those studies reporting that an irregular prolonged photoperiod increases the frequency of sexually formed embryo sacs in facultative apomictic grass species, a similar experimental setup was designed for the facultative apomictic herb *Ranunculus auricomus*. The *Ranunculus auricomus* complex (*R. auricomus* agg.) is a suitable and well-established model system for exploring reproduction modes and the mechanisms that trigger evolution of sex and apomixis (Nogler, 1984; Hörandl et al., 2009; Pellino et al., 2013). The complex comprises diploid sexual and polyploid facultative apomictic taxa that colonize a broad range of habitats between the arctic zone and the Mediterranean (Jalas and Suominen, 1998; Hörandl et al., 2009). The apomeiotic embryo sac development in *R. auricomus* starts from an unreduced somatic cell in the nucellus (apospory) while in the sexual pathway meiosis produces a megaspore tetrad, from which the functional megaspore develops into a reduced embryo sac. In both cases, the mature embryo sac is of the 7-celled, 8-nucleate *Polygonum* type which is typical for the majority of angiosperms (Nogler, 1984; Hojsgaard et al., 2014a). The unreduced egg cell develops parthenogenetically into an embryo, which is a clone of the mother plant. However, fertilization of polar nuclei by sperm cells is necessary for proper endosperm formation and development of functional seed (pseudogamy; e.g., Izmaiłow, 1996; Hörandl, 2008; Hojsgaard et al., 2014a).

Three different clonal progenies derived from the hexaploid (allopolyploid) natural hybrid *Ranunculus carpaticola × cassubicifolius* were investigated in the present experiment. This apomictic hybrid lineage has been naturally originated from the sexual diploid progenitor *Ranunculus carpaticola* Soó, distributed in the Carpathians, and the sexual autotetraploid *Ranunculus cassubicifolius* W. Koch from the northern-pre-Alps, during a hybridization event with subsequent genome duplication c. 70,000 years ago (Paun et al., 2006b; Pellino et al., 2013; Hojsgaard et al., 2015) and is known for facultative sexual/apomictic reproduction (Hojsgaard et al., 2014a). The higher frequencies of facultative sexuality in *R. auricomus* compared to *Boechera* (Aliyu et al., 2010), even under standard conditions, turn *Ranunculus* into a highly suitable model system for studying reproduction mode variation. Aposporous apomictic species can differ in their response to environmental factors (Houliston et al., 2006). Consequently, we investigated clonal progenies from three different genotypes (and source populations) from the same hybrid lineage. To entangle effects of prolonged photoperiods on apomeiosis and parthenogenesis, we analyzed mode of reproduction at two developmental stages, directly after megasporogenesis and at the seed stage. Under assumptions of the oxidative stress initiation hypothesis we would expect an effect of oxidative stress mainly during and directly after meiosis, resulting in increased proportions of functional megaspores.

Concomitantly, a targeted metabolomics analysis (HPLC–DAD) of phenolic metabolites in flower buds at the megasporogenesis stage was aimed at exploring (1) if the differing photoperiod treatment affected the patterns of detectable metabolites, and (2) if increased accumulation of a specific metabolite class or subclass of secondary, or as they are lately called, specialized metabolites, correlated with the treatment adding evidence to their suspected contribution as antioxidants to increased stress tolerance (Nakabayashi and Saito, 2015). For this purpose, the sole acquisition of UV spectra can be sufficient because it specifically allows detecting phenolic metabolites including the previously mentioned phenylpropanoid polyamine conjugates (Fellenberg et al., 2009; Gómez-Romero et al., 2010), all of which are renown *in vitro* antioxidants (Nakabayashi and Saito, 2015). Furthermore, the metabolic profiling was to explore (3) how the extent of reproductive phenotype switching compares to the detectable metabolic reprogramming. The targeted metabolic profiling detects many more metabolites besides phenolics.

## Materials and Methods

### Plant Material and Genotyping

Clone-mates of three populations were used for the experiment, i.e., offspring from three apomictically reproducing hexaploid *Ranunculus carpaticola × cassubicifolius* mother plants (originally collected from three sites in the western Carpathians, Slovakia, see Supplementary Table S1) was raised from seeds (Hörandl, 2008). Plants were pre-cultivated in pots under identical environmental conditions in the Göttingen University Botanical garden. Genotyping of clonal progeny was performed with nuclear microsatellite primers derived from an RNAseq dataset (Pellino et al., 2013). Loci including simple sequence repeats (SSRs) were detected using the program SciRoKo (Kofler et al., 2007), and amplified using PCR-primers (Supplementary Table S2) constructed in Primer3 (Untergasser et al., 2007). Leaves were sampled and dried in silica gel before DNA extraction with a DNeasy-96 Plant Kit (Qiagen, Hilden, Germany) following manufacturer’s recommendations. Multiplex PCR reactions were conducted in 25 μl reaction volumes containing: BIOMIX (12.5 μl); 10 μM F primer (0.2 μl); 10 μM R primer (1.0 μl); HEX or FAM CAG-Primer (1.0 μl); 1.0 μl of template DNA. Primers and all other PCR reagents were provided from Eurofins MWG Operon (Ebersberg, Germany). PCR reaction was conducted in BioRad T100^TM^ Thermal Cycler under following conditions: initial denaturation 94°C/10 min; 14 × [denaturation 94°C/60 s; annealing 62°C+0.5°C per Cycle 90 s; extension 72°C/60 s]; 35 × [denaturation 94°C/30 s, annealing 55°C/30 s, extension 72°C/30 s]; final extension 72°C/60 s; and storage at 4°C. PCR products were diluted in order to equalize their concentrations and mixed (volumes per line) with 85 μl Formamide (HiDi) and 0.7 μl size standard (GENSCAN 500 ROX; Applied Biosystems), denatured for 3 min at 92°C, and run on an automatic capillary sequencer Genetic Analyzer 3130 (ABI PRISM). Electropherograms were scored with GeneMarkerV2.4.2 (SoftGenetics LLC) and exported as binary matrix of presence/absence of alleles to characterize multilocus genotypes. Since up to six alleles occurred in some loci within a genotype, we could not identify allele dosage configurations in detail. The SSR profiles were subjected to neighbor-joining analysis (Supplementary Figure S1) based on Jaccard similarity index in FAMD (Schlueter and Harris, 2006). Branch support values are derived from majority consensus tree from 1000 bootstrap replicates. The resulting tree was visualized in FigTree v1.4.2 (Rambaut, 2009). This study ascertained that the three progenies cluster together and represent each maternal genotypes. Most individuals of one progeny (clone-mates) shared identical genotypes, only one to three individuals per clone had slightly different genotypes derived from SSR allele variants (see Supplementary Figure S1). Voucher specimens were deposited after the experiment in the herbarium of the University of Göttingen (GOET).

### Growth Chamber Setup

Preliminary experiments revealed that exposure to winter conditions is essential for flower induction. By end of February 2014, sprouting plants were transferred into growth chambers. From each clone eight clone-mates were grown with 10 h photoperiod (control plants) and 16 h photoperiod (stress treatment), respectively (see also Supplementary Figure S1). The treatment group received an additional short photoperiod of 0.5 h in the middle of the dark period to increase stress (16.5 h). These conditions were chosen based on preliminary experiments in attempts to assess the ideal photosynthetically active photon flux density (PPFD) and photoperiod duration conditions that still facilitated unimpeded floral and seed development of the *R. auricomus* clones in the available climate chambers. We refrained from varying light intensities for two reasons: first, the plants are forest understory plants, and hence pre-adapted to rather low light intensities; second, the technical conditions of the climate chambers limited our options of optimizing light intensities. The presented experiment was performed in climate chambers that were equipped with conventional gaseous discharge lamp technology. Its well-known disadvantages for plant culture, such as the inability to control the wavelength spectrum and the high radiant heat emission when delivering high light intensities, amongst others, also applied to this study. As a consequence, the available conditions had to be modified such that the treatment and control plants were still in sufficient good conditions to allow performing the measurement of all desired parameters.

The temperature was kept constant at 18°C in both chambers and relative humidity was set at 60%. The PPFD, photoactive radiation (PAR) in a more wide sense, was c. 250 μmol m^-2^s^-1^ (measured at shoot tips). The used light intensities reflect those in the understory and margins of forests, the natural habitat of the clone mother plants in Slovakia (Paun et al., 2006a). The control photoperiod was set a bit shorter than the natural day length of 12 h at the end of March/begin of April, because the technical setup of the chambers with conventional plant growth lamps did not allow the simulation of natural dusk and dawn periods with lower light intensity and changing wavelength spectra. To delimit radiation to the predominant stress factor, we instead increased photoperiod differences between stress and control. The plants grew in 31 plastic containers in conventional garden soil and were rotated regularly within chambers.

### Sporogenesis, Apospory, and Embryo Sac Development

Ovules were screened for the abundance of aposporous initial cells and functional meiotic megaspores (Hojsgaard et al., 2014a). For these embryological studies several buds per individual were harvested in early developmental stages and fixed in FAA (formalin : acetic acid : ethanol : dH_2_O; 2 : 1 : 10 : 3.5) for at least 24 h and maximal 48 h and then stored in 70% ethanol (Hojsgaard et al., 2014a). The perianth of the flower buds was removed and the gynoecium was cleared in methyl salicylate. Material was first dehydrated in three steps of 30 min incubation in 1 ml of 95% ethanol and two times in 100% ethanol, and then incubated for 30 min in 300 μl of upgrading series of methyl salicylate diluted in ethanol (25, 50, 70, 85%) and finally in 100% methyl salicylate (Young et al., 1979, with modifications). Ovaries (pistils) were dissected and mounted in methyl salicylate on glass slides. In *Ranunculus* each ovary contains one ovule. Female sporogenesis and early stages of sexual or aposporous gametophyte development were analyzed with differential interference contrast (DIC) in a light transmission microscope (Leica DM5500B with DFC 450 C camera, LAS V41 Software, Leica Microsystems, Wetzlar, Germany). All ovaries per bud were screened but just the clearly interpretable ovules were used for the evaluation (Van Baarlen et al., 2002).

Observations were evaluated in two classes (1) Pure sexual ovules: without any sign of apospory, but with a functional meiotic megaspore or one young sexual embryo sac (Supplementary Figure S2A), (2) Asexual or mixed ovules: containing an aposporous initial cell or one young aposporous embryo sac without or with additional meiotic products (megaspore or sexual embryo sac, Supplementary Figures S2B,C). Meiotic products abort sooner or later in such mixed ovules because the aposporous initials grow faster and take over the development (Hojsgaard et al., 2013, 2014a). We further did not evaluate separately ovules without any embryo sac development (i.e., when both pathways were not functional) which also occur regularly in *R. auricomus*.

### Reproductive Fitness/Seed-set

After we harvested several buds for biochemical analysis and microscopy, the remaining flowers of each plant were manually self-pollinated to allow for pseudogamy or self-fertilization (Hörandl, 2008). In the fruiting stage, five individual peduncles with collective fruits were bagged with porous plastic bags to avoid seed loss. Mature collective fruits were harvested, and the proportion of well-developed seeds (seed-set percentage) was determined per flower on individual and population level (after Hörandl, 2008); well-developed seeds were stored at 4°C until ploidy measurements (s. next section).

### Single Seed Flow Cytometric Seed Screening (ssFCSS)

Via flow cytometry the ploidy levels of the embryo and the endosperm in single seeds were measured and the reproductive pathway of each seed was calculated (Matzk et al., 2000). Single seeds were ground by two steel balls (Ø 4 mm) in a 2 ml Eppendorf cup with a Tissue Lyzer II (Qiagen, Hilden, Germany; 30 Hz s^-1^, time 7 s). Nuclear isolation and staining was conducted in two steps using Otto buffers (Otto, 1990; Doležel and Bartoš, 2005; Doležel et al., 2007). In the first step 200 μl Otto I buffer was added and mixed with the ground material for 30 s for the extraction of nuclei from the cells. The solution was then filtered (30 μm mesh, CellTrics^®^ Partec GmbH, Münster, Germany) into plastic tubes (3.5 ml, 55 mm × 12 mm, Sarstedt, Nümbrecht, Germany). In the second step 800 μl Otto II buffer [staining solution with 4′,6-diamidinophenyl-indole (DAPI)] was added to the filtrate and the solution was measured directly in a flow cytometer (CyFlow Space, Partec GmbH, Münster, Germany) in the blue fluorescence channel (UV LED, gain 366). The fluorescence intensity is proportional to the DNA content (ploidy) of the nuclei. The ratio of embryo : endosperm ploidy differs in sexual or asexual pathways (**Table 1**, Supplementary Figure S3). Mean values of the peaks were calculated with the program FloMax version 2.81 (Quantum Analysis GmbH, Münster, Germany) and peak indices (mean peak value of the embryo compared to mean peak value of the endosperm) were assessed (Microsoft Excel 2007).

**Table 1:** Seed formation pathways in hexaploid *Ranunculus carpaticola* × *cassubicifolius* plants assessed by flow cytometry.

Pathway		Embryo sac	Embryo (Em)	Endosperm (End)	Male gamete		Em + (End)	Expected PI
A	Sexual	Reduced	Zygotic	Fertilized	1 reduced		6C + (9C)	1.5
B	Apomictic	Aposporous	Parthenogenetic	Autonomous Endosperm	0		6C + (12C)	2.0
C	Apomictic	Aposporous	Parthenogenetic	Pseudogamous	1 reduced		6C + (15C)	2.5
D	Apomictic	Aposporous	Parthenogenetic	Pseudogamous	2 reduced	or 1 unreduced	6C + (18C)	3.0
E	Apomictic	Aposporous	Parthenogenetic	Pseudogamous		2 unreduced	6C + (24C)	4.0


The Peak indices (PI) of DNA content were calculated from the ploidy levels of the Embryo (Em) and the Endosperm (End) in seeds. PIs of the pathways A, C, D, and E were included in the evaluation. Pathway B is hypothetical as autonomous endosperm development was not reported for *Ranunculus auricomus*.

### Statistical Analysis of Reproduction Data

Microsoft Excel (2007) and IBM SPSS Statistics 22 (IBM Deutschland GmbH) were used. Data were tested for normal distribution by Kolmogorov–Smirnov and Shapiro–Wilk tests. The Levene test was used to check the homogeneity of variance. Percentages were arcsine transformed before statistical analysis. To test the influence of the treatment on mean sexual ovule and seed production for combined and single clones, analysis of variance (ANOVA) and pairwise *t*-tests were performed, respectively.

### Extraction and Analysis of Bud Secondary Metabolites

Buds (1–3) were collected between days 7 and 17 of the experiment, frozen in liquid nitrogen, and subsequently stored at -80°C. The frozen buds were ground with addition of a small amount of liquid nitrogen and transferred into disposable glass culture tubes (12 × 100, Duran GmbH, Mainz, Germany) with 5 ml MeOH (gradient grade, Th. Geyer GmbH and Co. KG, Renningen, Germany). After 48 h, the MeOH was evaporated (RVC 2-25 speedvac, Martin Christ Gefriertrocknungsanlagen GmbH, Osterode am Harz, Germany) and the residue dissolved in 5 ml of a mixture (3:2, v/v) of water (double distilled, GFL, Burgwedel, Germany) and ethyl acetate (p.a., Th. Geyer GmbH and Co. KG, Renningen, Germany). After intensive vortexing a phase separation was let to develop for 6 h. The upper ethyl acetate layer was removed and exchanged with new 2 ml. The procedure was repeated and the combined ethyl acetate fractions evaporated to dryness. The residue was dissolved in 100 μl MeOH:acetic acid (99:1, v/v) and transferred into glas autosampler vials with low-volume inlets (Macherey Nagel GmbH and Co. KG, Düren, Germany).

The HPLC system consisted of a Jasco PU-2085 Plus pump with a Jasco LG 2080-04S gradient former and DG 2080-54 degasser (Jasco Labor- und Datentechnik GmbH, Groß-Umstadt, Germany). The column was a Varian Polaris C18, 150 mm × 2 mm, 5 μm (Varian, Walnut Creek, CA, USA). The column oven was set to 40°C. Samples (10 μL) were injected with a Jasco A2-2059-SF Plus autosampler. A Varian Prostar UV diode array detector was used to record UV spectra from 220 to 600 nm. The system was controlled by Galaxy Chromatography Workstation 1.9.3.2 (Varian, Walnut Creek, CA, USA). The solvent gradient was binary with a constant flow rate of 0.2 ml min^-1^. Solvent A was 0.5% (v/v) *o-*phosphoric acid and solvent B MeOH. Solvent A was kept at 95% for 5 min and changed to 100% B in 95 min; 100% B was kept constant for 20 min.

### Statistical Analysis of Chemical Data

The analyzed chromatograms were extracted as maximum absorbance chromatograms, in which every peak was displayed by its optimum wavelength, and integrated using Galaxy Chromatography Workstation 1.9.3.2 (Varian, Walnut Creek, CA, USA). Peaks that were characterized by similar retention times and UV spectra were assigned to an identical variable. Peaks were considered between 10 and 117 min, but not lower than 10 min analysis times due to low separation quality.

Further statistics was performed with Primer 6 (Primer-E, Plymouth, UK). Samples were standardized by their totals. A two-dimensional ordination based on Bray–Curtis dissimilarity was created by non-metric multidimensional scaling (NMDS). Differences between treatments and clones were analyzed by ANOSIM (analysis of similarity). To test for genotype specific vs. individual responses of clone mates, a Mantel test (i.e., correlation between Jaccard genetic distances and Bray–Curtis dissimilarity of secondary metabolite profiles) of the analyzed individuals was calculated in PAST 2.17c (Hammer et al., 2001).

## Results

### Megasporogenesis and Aposporous Development

**FIGURE 1:**
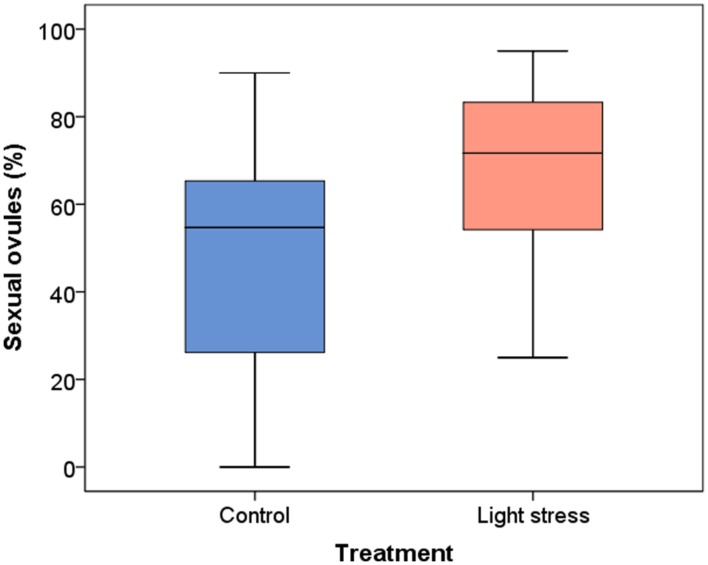
**Development of sexual ovules (%) in *Ranunculus carpaticola × cassubicifolius* plants grown in climate chambers under prolonged photoperiod (16.5 h, light stress) and shorter photoperiod (10 h, control plants).** *N* = 146 sexual ovules from 20 plants in the control and *n* = 181 sexual ovules from 18 plants in the stress treatment.

Two hundred and seventy one ovules in 20 control plants and 251 ovules in 18 stressed plants were investigated, on average 13–14 ovules per plant. Values are reported as means. For standard deviation and medians see **Figure 1**. In the extended photoperiod treatment (16.5 h) the proportion of sexual ovules was significantly increased compared to the control plants, from 47.9 to 66.5%. The mean difference was significant when data for all three clones were combined (*P* = 0.021, **Figure 1**). Sexual ovule production variation between individual plants was higher in the 10 h period compared to the 16.5 h photoperiod.

**FIGURE 2:**
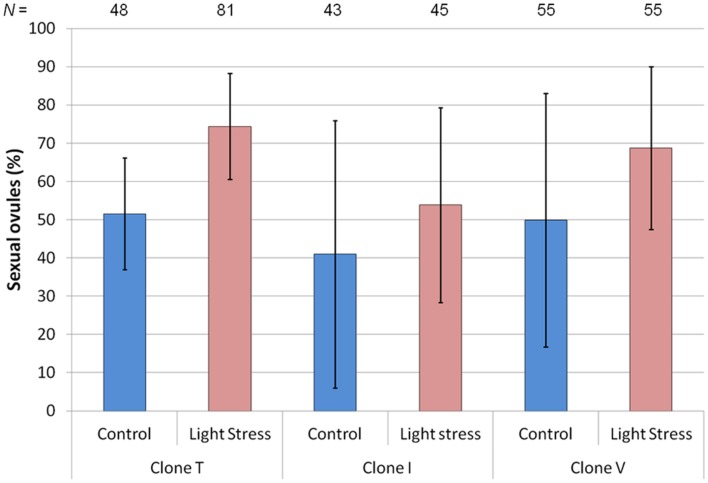
**Mean percentage of sexual ovules for three hexaploid *Ranunculus carpaticola × cassubicifolius* clones (T, I, and V) grown in climate chambers under enhanced photoperiod (16.5 h) and shorter photoperiod (10 h).** Error bars show standard deviation. Differences between treatments are significant for clone T (*P* < 0.01) but not for the other two clones (*P* > 0.20). *N* = number of sexual ovules.

The extended photoperiod increased the proportion of sexual ovules (**Figure 2**), but only clone T’s increase was highly significant (*P* = 0.008). The mean percentage of sexual ovules in this clone was 51.6% (10 h) and 74.4% (16.5 h), while for clone V’s mean percentages of sexual ovules increased from 49.9 to 68.8%, and for clone I from 41.0 to 53.9%. However, a high variance in both treatments led to non-significant differences for clone V (*P* = 0.264) and I (*P* = 0.553).

### Seed-set

The mean seed-set throughout all three clones (for more details see Supplementary Figure S4) was 42.1% well-developed seeds in the 16.5 h-photoperiod vs. 37.9% in the 10 h-photoperiod (*P* = 0.22). Seed-set success rates increased under prolonged photoperiods differently between clones. The effect was especially pronounced in clone T. Plants of this clone developed under the prolonged photoperiod significantly more viable seeds (42.7%) than in the control (33.0%; *P* = 0.04). The effect was less pronounced in clones V and I, 46.8% compared to 44.1% (*P* = 0.65), and 36.6% compared to 36.5% (*P* = 0.93).

### Flow Cytometric Seed Screening – Pathways of Seed Formation

In total 2056 seeds were analyzed and 1946 measurements, 949 seeds of the 10 h-photoperiod and 997 seeds of the 16.5 h-photoperiod could be used to assess the influence of the light treatment on the reproduction mode. Typical peak indices (PI) were 1.5 for sexual seeds (reduced embryo sac, zygotic embryo, one reduced male gamete contributes to the endosperm: two maternal nuclei + one paternal nucleus) and 2.5 for apomictic seeds [unreduced embryo sac (ES), parthenogenetic embryo, one reduced gamete contributes to endosperm] or 3.0 (unreduced ES, parthenogenetic embryo, one unreduced gamete or two reduced gametes contribute to endosperm; **Table 1**, pathways A, C, D). In a few seeds, ploidy counts resulted in a peak index of 4.0 (pathway E, **Table 1**) which differs from pathway D by the contribution of two unreduced male gametes to the endosperm, but otherwise is an apomictic pathway.

An apomictic pathway with autonomous endosperm development would result in a PI of c. 2.0 (pathway B, **Table 1**) but this pathway was not reported for *R. auricomus* to date. We excluded peak indices between 1.8 and 2.1 due to uncertainty of interpretation (4.7% of all measurements). Data with peak indices >4.0 (0.4% of all measurements) were probably due to putative endosperm endopolyploidy and therefore not taken into account.

**FIGURE 3:**
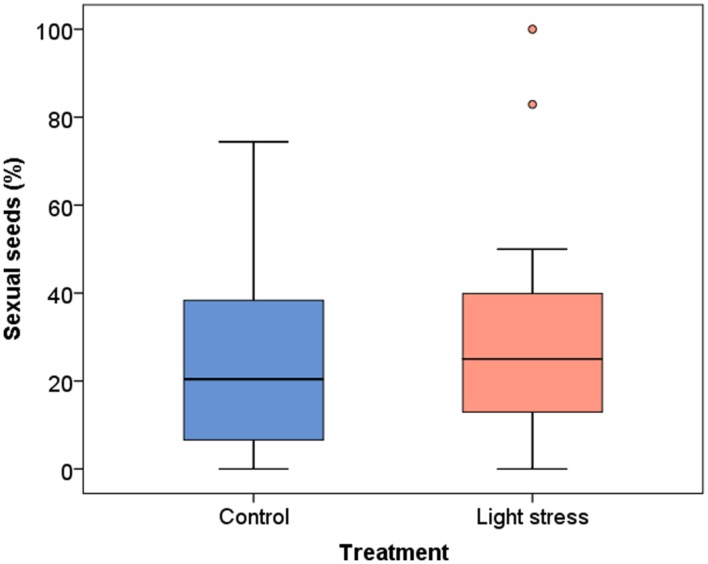
**Influence of the treatment on the production of sexual seeds (%) in *Ranunculus carpaticola × cassubicifolius* plants grown in climate chambers under prolonged photoperiod (16.5 h, *N* = 224) and shorter photoperiod (10 h, *N* = 257)**.

The mean proportion of sexually formed seeds in all three clones was slightly higher in the stress treatment (29.6% ± 23.7 *SD*, median 25.0%,) compared to the control plants (24.2% ± 20.1 *SD*, median 20.4%) but the difference was not significant (*P* = 0.336; **Figure 3**). The variability was high in both treatments, as single plants show 0 to 74% sexual seeds in the control and up to 100% in the stress treatment (**Figure 3**). The relative production of sexual vs. asexual seeds was enhanced in two clones (T and I) after the prolonged photoperiod, whereas clone V showed a slight decrease (Supplementary Figure S5). The differences between the treatments were not significant (*P* > 0.10).

### Secondary (Specialized) Metabolite Patterns in Flower Buds

**FIGURE 4:**
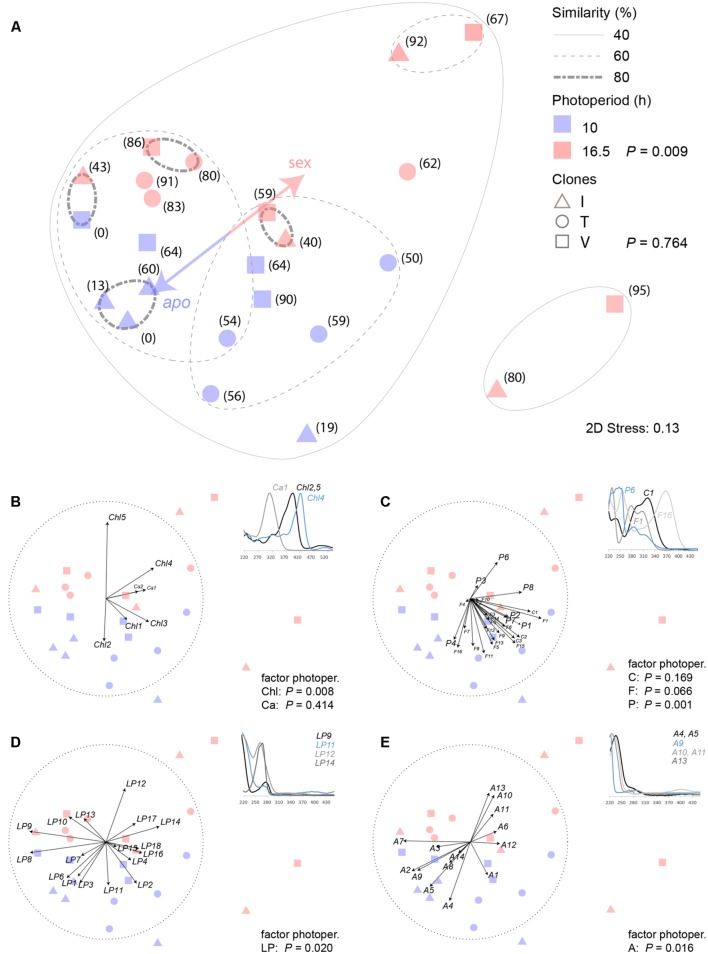
**Similarity analysis of HPLC-DAD detectable secondary metabolites in closed buds of three hexaploid *Ranunculus carpaticola × cassubicifolius* clones (I, V, T) exposed to a 10 and 16.5 h photoperiod. (A)** NMDS of Bray–Curtis dissimilarity of standardized peak area patterns of 66 metabolites; bracket numbers are percentages of sexual ovules formed in one flower; *P-*values (ANOSIM) inform about similarity and dissimilarity of the indicated factors photoperiod (*N* = 12) and clone (*N* = 8); **(B–D)** variable contributions of different metabolite classes illustrated by 3–4 representative UV spectra (220–450 nm) of **(B)** chlorophyll degradation products (Chl) and carotenoids (Ca), **(C)** cinnamic acids (C), flavonoids (F) and other polar phenols or alkaloids (P), **(D)** lipohilic phenols, alkaloids or other more unsaturated metabolites (LP), and **(E)** aliphatic and more saturated metabolites (A). To correlate to mode of reproduction in the early developmental stage, percentage of sexual ovules of each individual was included as one variable in the NMDS ordination.

HPLC–DAD analyses resulted in detecting 66 metabolites (Supplementary Table S3), the majority of them probably secondary. The assignments and, in some cases, tentative identification were based on a unique combination of retention time and UV spectra (Supplementary Methods 1). ANOSIM analyses of the total data set revealed that differential photoperiod treatment caused a dissimilarity of the metabolic profiles (*P* = 0.009). A global *R* of 0.198 indicated that about one fifth of the analyzed metabolites contributed to the observed dissimilarity. If instead clone identity was used as grouping factor, no dissimilarity between the groups could be detected (*P* = 0.764). A NMDS plot based on Bray–Curtis dissimilarities illustrates this (**Figure 4A**).

In attempts to obtain more specific insights, the 66 detected metabolites were assigned to several distinct groups as far as the available information allowed to do this. **Figure 4B** illustrates the correlation (Spearman) of carotenoids (Ca) and chlorophyll degradation product (Chl) variables with the obtained ordination in the NMDS plot. The latter contributed significantly to the dissimilarity of the photoperiod duration treatment groups (*P* = 0.008, *R* = 0.238). In the 10 h-period, pheophytin A (Chl5) was formed, which changed to pheophorbide A (Chl2) in the 16.5 h period. These metabolites were tentatively identified based on published analyses and spectral data by Mangos and Berger (1997).

Cinnamic acids (C), flavonoids (F) and other polar phenolic metabolites (P) were subsumed within the second group that comprised the majority of potential phenolic antioxidants (**Figure 4C**). Neither the cinnamic acid derivatives (C, *P* = 0.169), among which UV spectra pointed to the presence of caffeic and ferulic acid (Supplementary Methods 1), nor flavonoids (F, *P* = 0.066), for which UV spectra suggested the presence of trihydroxyflavone, herbacetin, kaempferol, orientin, patulitrin, quercetin, rhamnetin, and vitexin glycoside structures (Mabry et al., 1970; Supplementary Methods 1), showed patterns that correlated with the photoperiod duration, quite in contrast to the unassigned phenols (P) (*P* = 0.001, *R* = 0.318).

The third group (LP) subsumes all those metabolites with UV spectra showing a more prominent absorption maximum at 280 nm at least. Lipophilic phenols, alkaloids, or more highly unsaturated aliphatic secondary metabolites represent potential candidate structures (**Figure 4D**). Again, some metabolites supported the different photoperiod treatment (*P* = 0.02, *R* = 0.153).

The fourth group (A) subsumes aliphatic compounds, most probably oxidized fatty acids derivatives (oxylipins) or terpenoids with the common characteristic of an absorption maximum at 250 nm or lower (**Figure 4E**). A small portion also supports the dissimilarity that was caused by the different treatments (*P* = 0.016, *R* = 0.168).

In summary, the extended photoperiod caused complex pattern changes in the detectable metabolites in the buds that, on one hand, correlated with the dissimilarity of the two treatment groups, and on the other hand, cannot be explained by an identical set of metabolites for each accession.

Analysis of similarity analyses were also performed for the factor clone but the results (not shown) indicated no dissimilarity for the individual clones I, T, and V. This was further confirmed by a Mantel test which revealed no significant correlation of genetic vs. secondary metabolite diversity among clones (*P* = 0.9) which is illustrated by a graphical plot of genetic distance vs. dissimilarity of secondary metabolites (Supplementary Figure S6).

## Discussion

The present study provides considerable evidence for a similar effect of photoperiod extension on reproduction mode in *R. auricomus* as revealed by the few previous studies carried out with grasses, specifically a slight increase in sexual ovule formation (Knox, 1967; Evans and Knox, 1969; Gupta et al., 1969; Saran and Dewet, 1976; Quarin, 1986). The light intensity in the chambers matched roughly outdoor light conditions in shaded or half shaded habitats typical for natural populations. The extended photoperiod in the stress treatment (16 h plus 0.5 h night break) was designed to simulate a moderate light stress, all other environmental factors being kept equal. Too extensive stress could cause strong detrimental effects. This effect can especially occur if radical-producing chemicals are infiltrated into the tissue. For instance, a study that applied this approach claimed that oxidative stress negatively correlates with meiosis in maize anthers (Kelliher and Walbot, 2012).

The facultative apomictic/sexual *Ranunculus* plants produced altogether significantly more sexual ovules with 16.5 h photoperiod compared to the 10 h light treatment. A similar effect was observed in facultative apomictic tetraploid *Paspalum cromyorrhizon* plants, where a prolonged photoperiod (14 h compared to 12 h) enhanced the percentage of ovules with embryo sac development from a reduced functional megaspore (Quarin, 1986). The authors assumed the light regime to be one of the environmental factors that influences reproduction mode in facultative apomictic/sexual *Paspalum*. In contrast to *Paspalum*, an evaluation of the percentage of sexual ovules at a later stage (mature embryo sac during flowering) was not possible via microscopy as in the genus *Ranunculus* ovules do not show multiple embryo sacs, and mature aposporous and sexual embryo sacs are phenotypically identical (Hojsgaard et al., 2014a).

The higher percentage of sexual ovules in the stressed plants resulted in a slightly higher mean frequency of sexual seeds compared to control plants, but the difference was not significant. Houliston et al. (2006) studied the influence of the photoperiod on the reproductive mode of facultative apomictic *Hieracium pilosella* and found no significant difference between a 14 and 16 h treatment in the germination rates of seeds. The results suggest that light stress just increased frequencies of functional megasporogenesis in *R. auricomus*, but had no direct effect on later embryo sac formation and seed development. But, a striking decrease of sexuality was observed between ovule and seed stage. The frequency of sexual ovules was twice as high as the frequency of sexual seeds in both light regimes. A strong decline of the proportions of functional megaspores to proportions of well-developed embryo sacs, and finally to sexually formed seeds occurred also in the hexaploids of *R. auricomus* cultivated under equal garden conditions, while in diploid sexual species these proportions remained at the same level between the developmental stages (Hojsgaard et al., 2014a). A similar decline of sexual development between the developmental stages was observed by Hojsgaard et al. (2013) in apomictic *Paspalum malacophyllum*. In *R. auricomus*, aborting vs. functional phenotypes of sexual embryo sacs can be well-observed by microscopic investigation at the early stages (Hojsgaard et al., 2014a). The (2n) aposporous embryo sac development seems faster than the (n) meiotic one after the appearance and growth of one or several unreduced aposporous initial cell(s) simultaneously to the functional reduced megaspore in ovules where both pathways are functional. Survival of the aposporous sacs and abortion of the meiotic ones then results in higher frequencies of apomictic seeds. The decline of reduced megaspores/gametophytes can be enhanced by expression of deleterious recessive mutations in the growing gametophyte, resulting in abortion, while in unreduced gametophytes, recessive mutations would be masked (Hojsgaard and Hörandl, 2015). This trend seems to be recurrent for facultative apomictic species, where the asexual pathway often prevails probably due to the genetic or epigenetic disorders causing asynchronous development on the sexual pathway after activation of apomixis (Hojsgaard et al., 2013). As a consequence, possibly in our study the decline between the relative proportions of sexual megaspore formation to sexual seeds is influenced more by the competitive capacity of the apomictic pathway rather than by the light regime during gametogenesis and seed development. This supports the oxidative stress initiation hypothesis (Hörandl and Hadacek, 2013) which predicts that light stress affects only female meiosis, but has not relevant effects on further ovule and seed development.

All three progenies showed a considerable variation among clone-mates in frequencies of megasporogenesis vs. apospory. This result strongly supports epigenetic and transcriptional control mechanisms as the background for the phenotypic expression of apospory (Grimanelli, 2012; Schmidt et al., 2014). Allelic dosage effects as observed in Mendelian genetic studies (Nogler, 1984; Ozias-Akins and Van Dijk, 2007) obviously can be modulated by environmental influence. Methylation-based epigenetic variation does exist in genetically identical clone-mates and is sensitive to environmental stress, as shown in *Taraxacum* (Verhoeven et al., 2010). A possible contribution of secondary metabolites in this modulation of reproductive pathways is indirectly supported from gene expression studies. Transcriptome profiles of flowering buds of *Hypericum perforatum* revealed that most GO terms of differentially expressed genes between sexual and aposporous accessions were related to stress response, biotic and abiotic stimuli, DNA metabolic profiles, and signal transduction, but also to carbohydrate and lipid metabolic profiles, and to secondary metabolites (Galla et al., 2015). Strikingly, even cell-specific transcriptome profiles of laser-microdissected aposporous initials in *Boechera gunnisoniana*, compared to sexual *Arabidopsis thaliana*, revealed that several regulatory pathways differ between sexual and aposporous germlines, including also enrichment of spermidine and polyamine pathways (Schmidt et al., 2014). In *Ranunculus* a comprehensive gene expression profiling is not yet feasible due to the lack of an annotated reference genome (see Pellino et al., 2013). In this respect, this study attempts as a first step to find a covariance between mode of reproduction and secondary metabolite profiles under stress conditions.

Numerous studies document that secondary or specialized plant metabolites, especially phenolic ones, possess antioxidant properties *in vivo* and are thus associated with the plant’s response to abiotic stress (Tohge et al., 2013; Nakabayashi and Saito, 2015). In context with the oxidative damage hypothesis for meiosis, an assessment of such metabolite profiles in buds that have been formed on plants exposed to photoperiods of different length represented a feasible methodological approach to detect potential changes caused by the potentially resulting oxidative stress. ROS are usually short-lived but can cause complex tissue-wide oxidation when they are formed excessively and trigger uncontrolled oxidative chain reactions (Møller et al., 2007). As a result of the increased oxidation, the profiles of detectable metabolites were expected to show notable changes. These can be caused either by the formation of novel structures (oxidation products or novel biosynthesis) or loss due to polymerization resulting in non-detectable products. The investigated accessions comprised each four individuals from the three clones I, T, and V. In terms of detectable metabolites, the phenotypic variation within the three investigated clones was notable. Only three individuals in one clone (I) showed 80% similarity. These results support the hypothesis that this variation might be caused by epigenetic mechanisms as a consequence of variable micro-conditions which have to be expected when plants were grown in garden and not in growth chamber conditions. Exposure to winter frost conditions, however, was mandatory to obtain flowering plants in spring. Despite this unavoidable heterogeneity—all plants were of the same age and always kept in the same location before the experiment—the extended photoperiod still changed significantly the metabolite patterns (**Figure 4A**). If one takes a look at different metabolite classes (**Figures 4B–E**), their single contributions vary. Chlorophyll degradation products (Chl) provide a highly significant contribution to the two photoperiod treatments (**Figure 4B**); this is not unexpected as chlorophyll degradation is an oxidation process (Hörtensteiner, 2006). We suppose that the photosynthetically active green sepals of the buds were the major affected tissue that responded to the stress that was caused by the extension of the photoperiod. The contribution of carotenoids (Ca) was not significant (**Figure 4C**). These metabolites cause the yellow color of the petals (Grotewold, 2006). Consequently, their detectability could be low because of the early developmental stage of the petals in the buds, which could have varied between and within clones. Moreover, petals are often more or less aborted in apomictic *R. auricomus* plants (Hörandl et al., 2009). Phenylpropanoid acids (C) and flavonoids (F) did not add to the treatment group separation which somehow contradicts their attributed reputation as antioxidants (Nakabayashi and Saito, 2015). Similar to carotenoids, they might not yet be expressed at this early bud stage. Conversely, they added to variation within the single accessions that received the same photoperiod treatment, though (**Figure 4C**). A highly significant contribution, however, was added by non-assigned phenolic compounds (P, **Figure 4C**). Some could represent conjugates with other stress-related metabolites, such as phenylpropanoid polyamine conjugates (Fellenberg et al., 2009). The retention times of identified phenylpropanoids, however, suggest more the presence of non-conjugated phenylpropanoids. Unfortunately, many of these conjugates have been identified previously in strict LC–MS analyses, in which reporting of UV spectral data is usually omitted in contrast to reports on novel isolated plant metabolites. The more lipophilic group of phenolic metabolites (LP, **Figure 4D**) was comprised of mostly unknowns. It is well-known that the various forms of environmental stress can also induce or enhance non-phenolic secondary metabolites (Ramakrishna and Ravishankar, 2011). In this context, it was not totally unexpected that the last group, the aliphatic and more saturated metabolites (A, **Figure 4E**), contributed to the dissimilarity of the treatment groups similarly to the LP group. As the previously discussed group, A is also heterogeneous and most probably comprised of terpenoids, most likely of variously oxidized sesqui-, triterpenes, oxylipins, or oxidized fatty acid degradation products. The initial hypothesis predicted that the extended photoperiod predominantly affects the presumed antioxidants, cinnamic acids (C) and flavonoids (F). A lot of attention is paid to flavonoid and other phenols due to their diversity, ubiquity and documented *in vitro* bioactivity (Buer et al., 2010) in assumptions that the activity is mainly antioxidant (Nakabayashi and Saito, 2015). Other studies, however, point to the fact that anti- and pro-oxidant activities depend both on electron availability and metal catalysts of these electron transfers and are thus highly locally milieu-dependent (Chobot et al., 2013). Substantial experimental evidence points to the fact that the cellular redox signaling hub interacts complexly with the hormone signaling network at multiple levels, which synergistically not only controls plant growth and development but also modulates response to environmental stress (Considine and Foyer, 2014). However, only few components of this complex system are understood so far. Furthermore, the results obtained in this study point to a more systemic than a linear deterministic nature of the involved processes, in which specific quantitative changes of metabolite concentrations fail to explain the overall observed effect.

## Conclusion

We confirmed the hypothesis that a prolonged photoperiod, as a moderate environmental stress, can increase frequencies of ovules with functional megaspores of facultative apomictic plants, but it can fail affecting the seed development. The stress response in flowering buds is accompanied by changes of secondary metabolite profiles. These were not caused by the reputedly antioxidant flavonoids and phenylpropanoids, which might not yet be expressed at this early stage, but by unidentified phenols and other, partially non-phenolic compound classes. Potentially, oxidative stress, as a systemic response specific for the early developmental stage, could have affected megasporogenesis. So far, no comparative gene expression study is available on stress response of sexual and apomictic plants, or of facultative apomictic plants. In preparation of such more extensive investigations, this study confirmed the assumed covariance of modes of reproduction and of changes of secondary metabolite profiles under a controlled experimental setup for the *R. auricomus* model system, which represents the first herbal plant species ever investigated in this context.

## Author Contributions

SK, FH, and EH conceived and designed the study. SK, GB, ME, DH, and EH performed experiments and analyzed reproductive data, FH did the biochemical analyses, LH the genotyping. SK, FH, and EH wrote the manuscript with the support of all authors.

## Conflict of Interest Statement

The authors declare that the research was conducted in the absence of any commercial or financial relationships that could be construed as a potential conflict of interest.
