# Supplementary material for: Photoperiod Extension Enhances Sexual Megaspore Formation and Triggers Metabolic Reprogramming in Facultative Apomictic Ranunculus auricomus
Source: Front Plant Sci. 2016 Mar 8;7:278. doi: 10.3389/fpls.2016.00278 (PMC4781874; doi:10.3389/fpls.2016.00278)
Supplement: Supplementary file 1 [file Data_Sheet_1.DOCX]

Supplementary Material

Photoperiod extension enhances sexual megaspore formation and triggers metabolic reprogramming in facultative apomictic *Ranunculus auricomus*

Simone Klatt, Franz Hadacek, Ladislav Hodač, Gina Brinkmann, Marius Eilerts, Diego Hojsgaard, Elvira Hörandl*

*** Correspondence:**

Elvira Hörandl

elvira.hoerandl@biologie.uni-goettingen.de

# Supplementary Figures and Tables

## Supplementary Figures


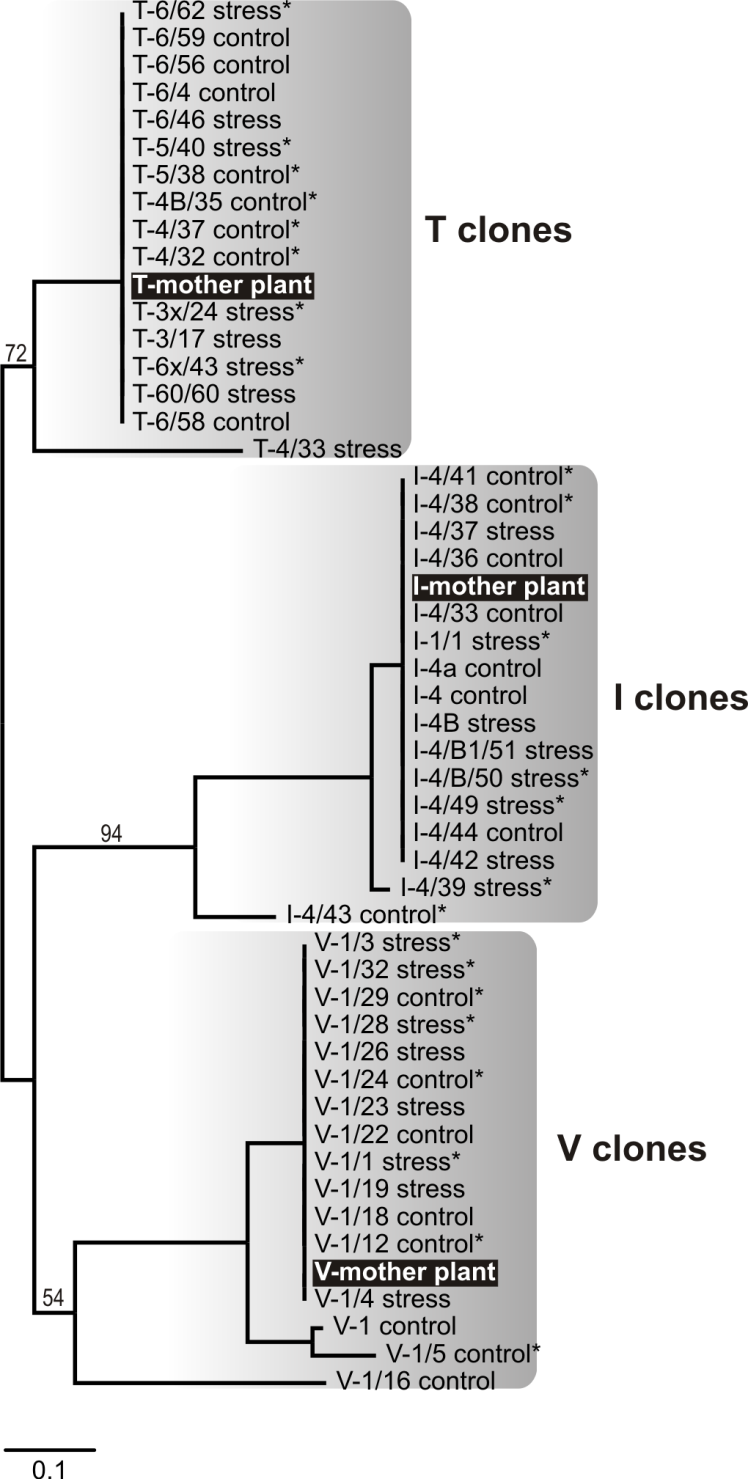


**Supplementary Figure S1.** Neighbor-joining tree derived from SSR data. Three *Ranunculus carpaticola × cassubicifolius* mother plants (i.e., T, I, V) and their clonal progeny were analyzed. Individuals used for analyses of secondary metabolites are marked with an asterisk.


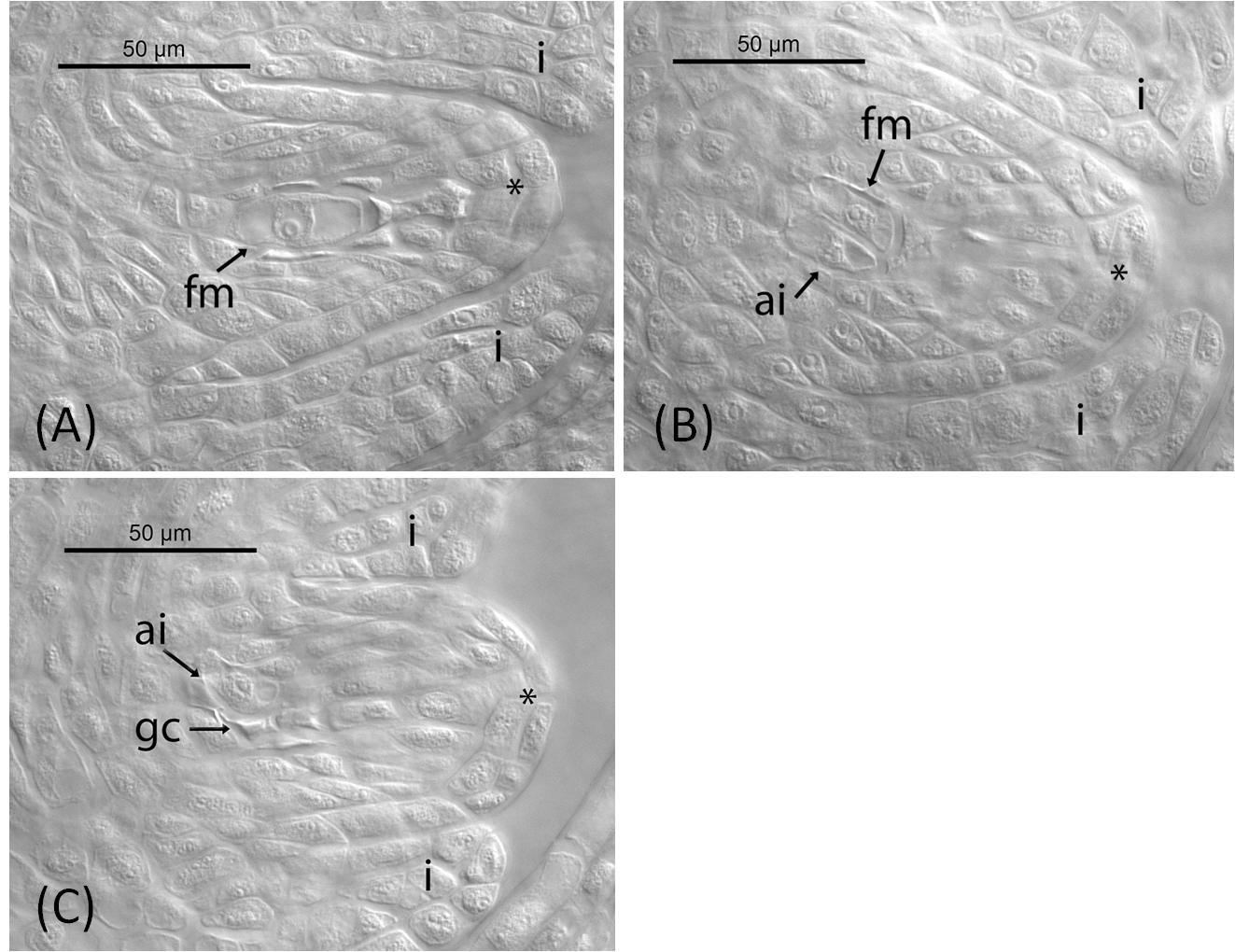


**Supplementary Figure S2.** Sexual, asexual and mixed ovules produced by facultative apomictic *Ranunculus auricomus*. (A) sexual ovule with one functional megaspore containing a big nucleus with one visible nucleolus and two visible vacuoles, adjacent meiotic cells in the direction of the micropyle are aborting; (B) ovule with both sexual/asexual pathways, one functional megaspore in a line with aborted germline cells and one aposporous initial cell of similar size as the megaspore; (C) asexual ovule with aposporous initial cell adjoining the row of germline cells (four meiotic products, aborting); fm, functional megaspore; ai, aposporous initial cell; gc, germline cells; i, integument; *, micropylar pole. Bars, 50 µm.


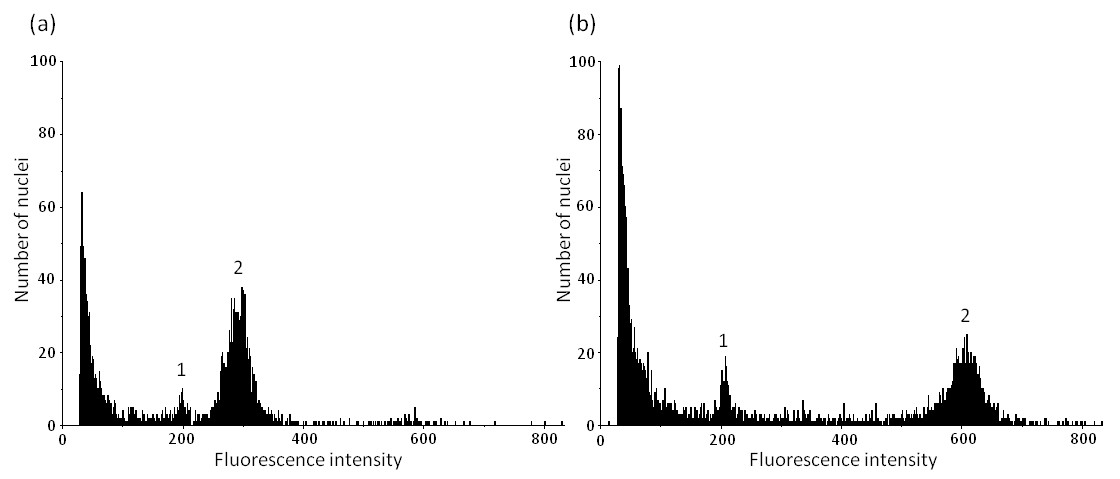


**Supplementary Figure S3.** Flow cytometry histograms of a sexual seed (a) and an asexual seed (b) produced by facultative sexual / apomictic *Ranunculus auricomus* plants (two plants of clone I). The fluorescence intensity (relative indication) reflects the relative DNA content of the measured nuclei. Peak 1, nuclei from the embryo tissue; peak 2, nuclei from the endosperm tissue. The ratio of 1 : 1.5 (embryo DNA content : Endosperm DNA content) indicates a sexual seed. The ratio of 1 : 3 was evaluated as asexual seed.


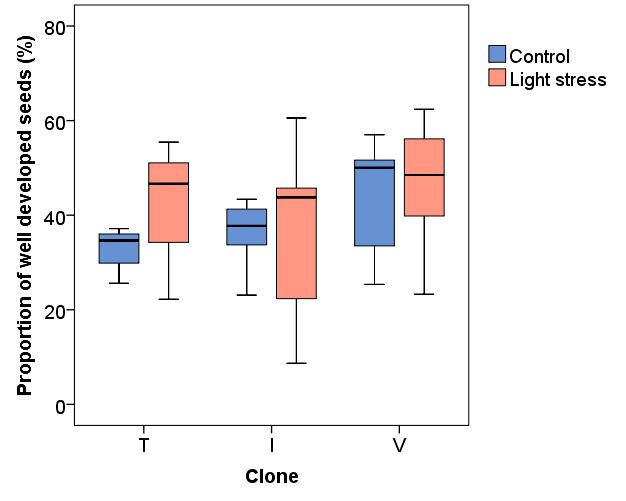


**Supplementary Figure S4.** Proportions of well developed seeds (%) per flower for three hexaploid *Ranunculus carpaticola × cassubicifolius* clones (T, I, and V) grown in climate chambers under enhanced light period (16.5 hours, stress, red boxes) and shorter light period (10 hours, control, blue boxes). N = 8 plants per clone and treatment.


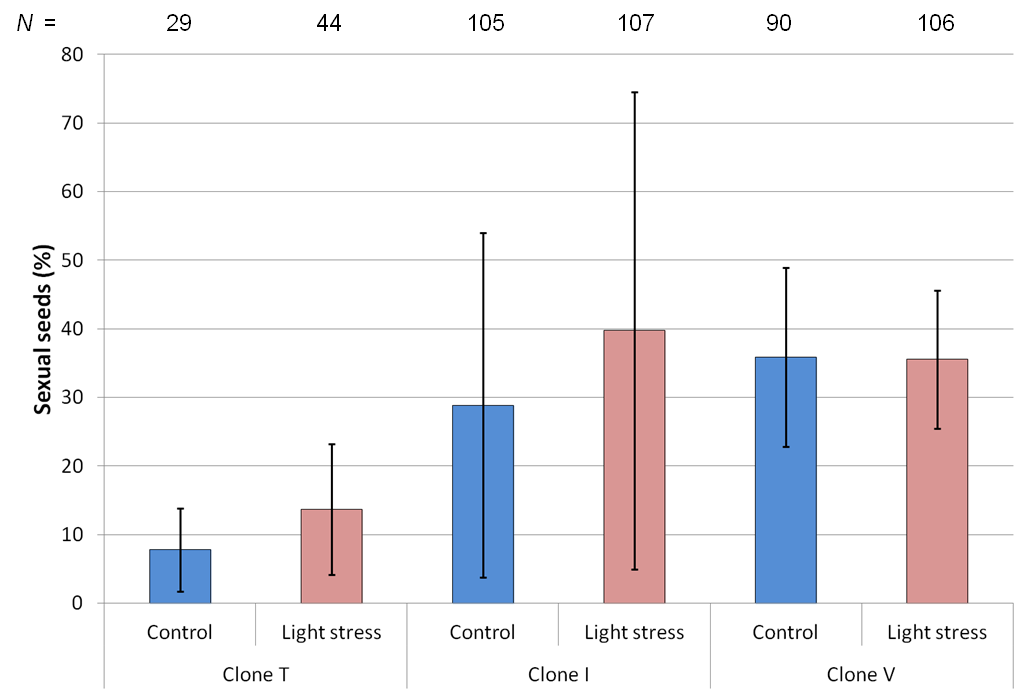


**Supplementary Figure S5.** Mean percentage of sexual seeds for three hexaploid *Ranunculus carpaticola × cassubicifolius* clones (T, I, and V) grown in climate chambers under enhanced light period (16.5 hours, red bars) and shorter light period (10 hours, blue bars). Error bars show standard deviation. *N* = number of sexual seeds. Differences between treatments are not significant (clone T: *P* = 0.165, clone I: *P* = 0.397, clone V: *P* = 0.937).


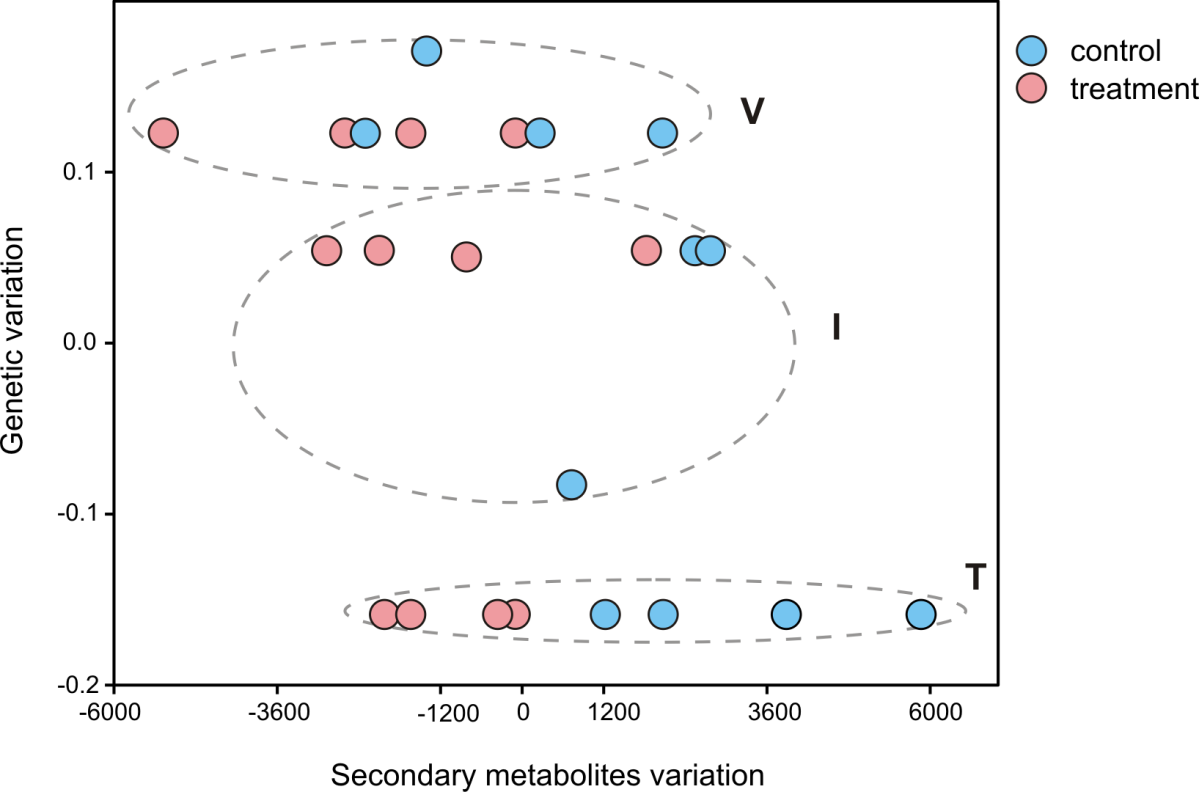


**Supplementary Figure S6.** Variation of secondary metabolites (strongest PCA axis) and genetic variation (strongest MDS axis), illustrating that the former is not genotype-specific, but follows the treatments. The individual rather than genotype specific response was visualized as a plot of scores obtained from two different ordination techniques. First, a NMDS was conducted on a matrix of genetic (Jaccard) similarities among individuals. The resulting ordination revealed first component to separate best among the I, T and V clones. Second, a PCA was conducted on the secondary metabolites, where the third component (9% of the total variability) separated best between treated and control clones. Third, the genetic variation (= scores on the 1st NMDS component) and the secondary metabolites variation (= scores on the 3rd PCA component) were plotted against each other in a scatter plot. All analyses were conducted in PAST 2.17c (Hammer *et al.*, 2001).

Hammer, Ø., Harper, D. A. T., Ryan, P. D. (2001). PAST: Paleontological Statistics software package for education and data analysis. Palaeontologia Electronica 4: 1-9.

## Supplementary Tables

**Supplementary Table S1.** Habitats and collection date of the three mother plants from the hexaploid natural hybrid *Ranunculus carpaticola × cassubicifolius*. Clonal offspring from the individuals T, V and I produced in 2007 (Hörandl, 2008) was used in the experiment.

| **Population** | **Abbreviation of original population in earlier papers** | **Location/Habitat of mother plant** | **Collectors and herbarium vouchers** |
| --- | --- | --- | --- |
| T | TRE  ^1^Paun *et al.* (2006a,b), Hörandl *et al.* (2009), Pellino *et al.* (2013) | C. Slovakia, Strážovské vrchy (near Trenčin), between Kubra and Kubrica, close to the bus stop Kyselka (margin of *Carpinus* forest and meadow) | Mládenkova, Paun & Hörandl C29, 30.4.2004 (SAV) |
| V | VRU2  ^1^Paun *et al.* (2006a,b), Hörandl *et al.* (2009), Pellino *et al.* (2013) | C. Slovakia, Turčianska kotlina, Vrútky-Piatrová, behind the cottage (meadow) | Mládenkova, Paun & Hörandl C35, 1.5.2004 (SAV) |
| I | IVAC  Hörandl *et al.* (2009), ^1^Hörandl & Greilhuber (2002), ^1^Paun *et al.* (2006a,b) | C. Slovakia, Liptovská kotlina, Ivachnová (forest and meadow) | Hörandl, 8492, 1.05.1998 (WU) |

^1^ under the previous name *R. carpaticola* (hexaploid)

Hörandl, E. (2008). Evolutionary implications of self-compatibility and reproductive fitness in the apomictic *Ranunculus auricomus* polyploid complex (Ranunculaceae). *Int. J. Plant Sci.* 169, 1219–1228. doi: 10.1086/591980

Hörandl, E., and Greilhuber, J. (2002). Diploid and autotetraploid sexuals and their relationships to apomicts in the *Ranunculus cassubicus* group: insights from DNA content and isozyme variation. *Plant Syst. Evol.* 234, 85-100. doi: 10.1007/s00606-002-0209-x

Hörandl, E., Greilhuber, J., Klímová, K., Paun, O., Temsch, E., Emadzade, and K., and Hodálová, I. (2009). Reticulate evolution and taxonomic concepts in the *Ranunculus auricomus* complex (Ranunculaceae): insights from analysis of morphological, karyological and molecular data. *Taxon* 58, 1194–1215.

Paun, O., Stuessy, T. F., and Hörandl, E. (2006a). The role of hybridization, polyploidization and glaciations in the origin and evolution of the apomictic *Ranunculus cassubicus* complex. *New Phytol.* 171, 223–236. doi: 10.1111/j.1469-8137.2006.01738.x

Paun, O., Greilhuber, J., Temsch, E. M., and Hörandl, E. (2006b). Patterns, sources and ecological implications of clonal diversity in apomictic *Ranunculus carpaticola* (*Ranunculus auricomus* complex, Ranunculaceae). *Mol. Ecol.* 15, 897–910. doi: 10.1111/j.1365-294X.2006.02800.x

Pellino, M., Hojsgaard, D., Schmutzer, T., Scholz, U., Hörandl, E., Vogel, H., and Sharbel, T. F. (2013). Asexual genome evolution in the apomictic *Ranunculus auricomus* complex: examining the effects of hybridization and mutation accumulation. *Mol. Ecol.* 22, 5908–5921. doi: 10.1111/mec.12533

**Supplementary Table S2.** Characteristics of SSR markers developed for *Ranunculus carpaticola × cassubicifolius*.

| **Locus** | **Primer sequences (5′–3′)** | **T_a_^1^ (°C)** | **Repeat motif** |
| --- | --- | --- | --- |
| LH03 | F: GTCCGATCTGCGATTCCGAT | 55 | (GAT)_9_ |
|  | R: TCGGAGTCGATTCAAGATTTCGA | 55 |  |
| LH08 | F: GGAGGATATGAGCGGTTCGA | 54 | (CA)_8_(TA)_7_ |
|  | R: TATGATGCGTATGGGCGGAG | 55 |  |
| LH09 | F: TTATACGTGACCATCCGCCG | 55 | (TG)_6_(CG)_4_ |
|  | R: CATTTTCAATGGTGCGAATACGA | 53 |  |
| LH11 | F: CCAACGGACACTGCTCTTCT | 55 | (TC)_18_ |
|  | R: TGCTACTCAACCTTGAACTCGA | 54 |  |
| LH12 | F: TTTAAGGGCATGCGACCCAT | 55 | (TC)_18_ |
|  | R: TCCCAGGGACAGAACTTTGC | 55 |  |

^1^Annealing temperature.

**Supplementary Table S3.** HPLC−DAD analyses of buds, clone individual identity, number of analyzed buds (pooled), peak integration values (100 = not detected); double-click to open the complete table in Excel format.

# Supplementary Methods

**Supplementary Methods S1.** UV spectra and retention times of scored metabolites (HPLC−DAD analyses). **
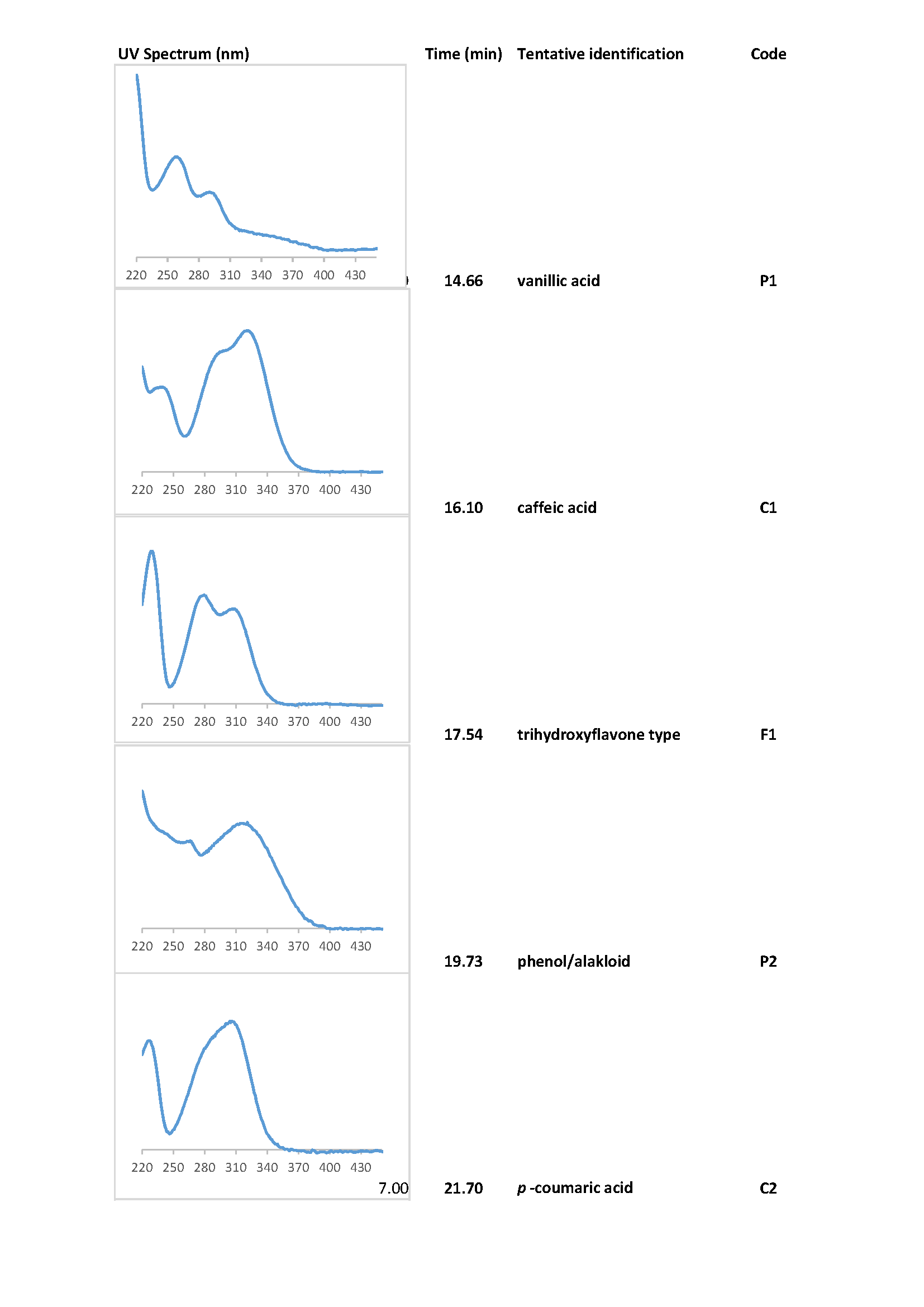
**

**
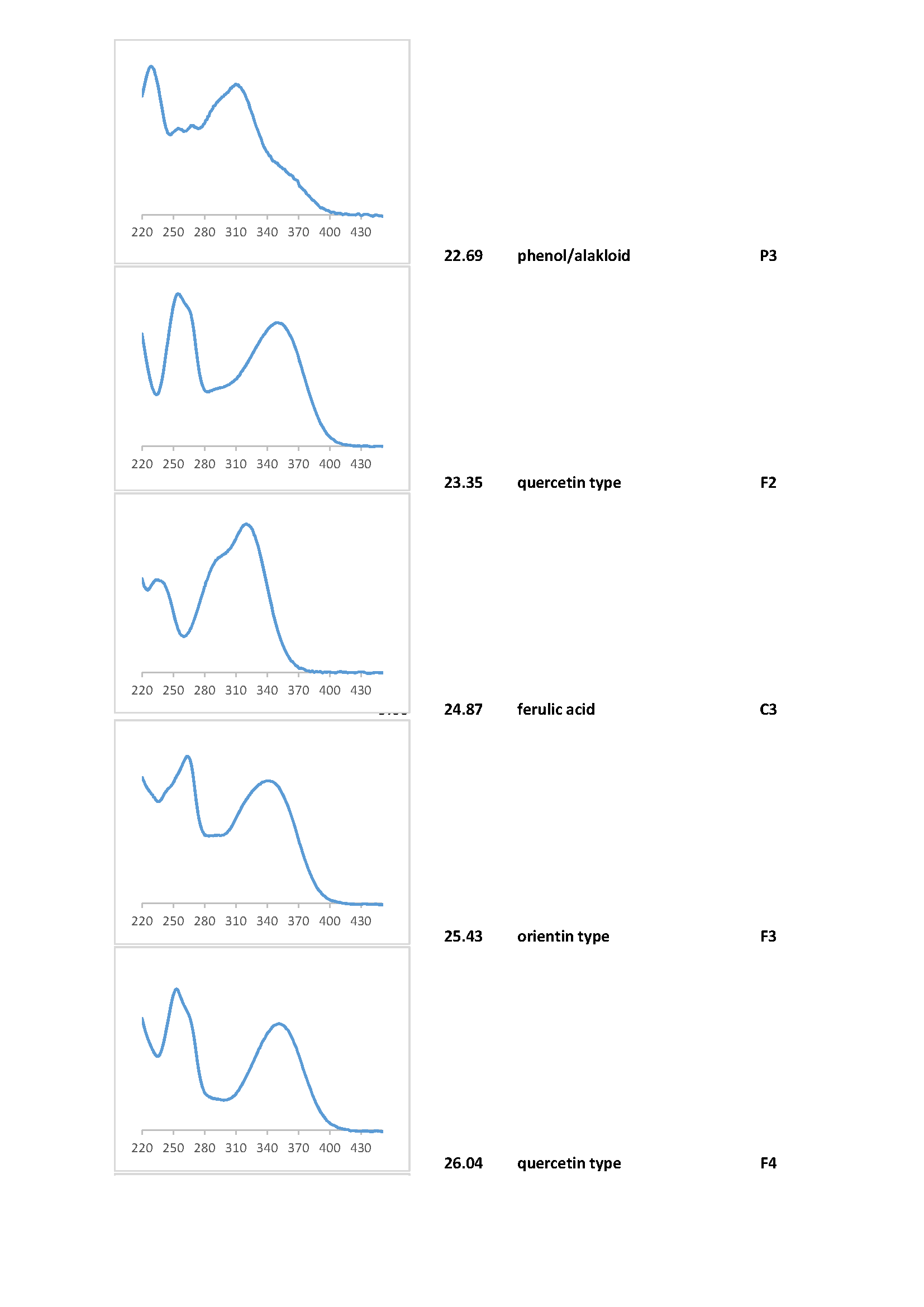
**

**
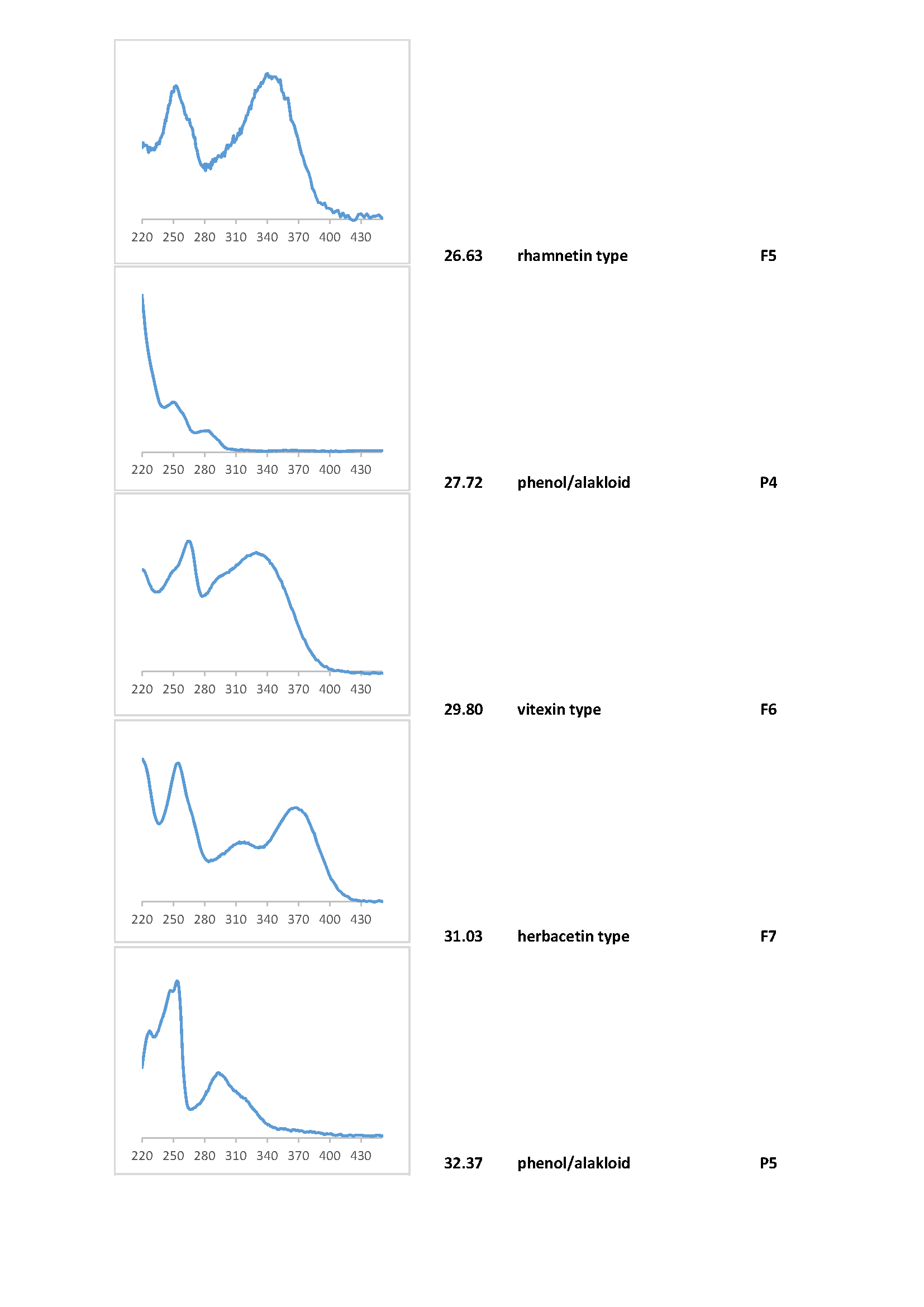
**

**
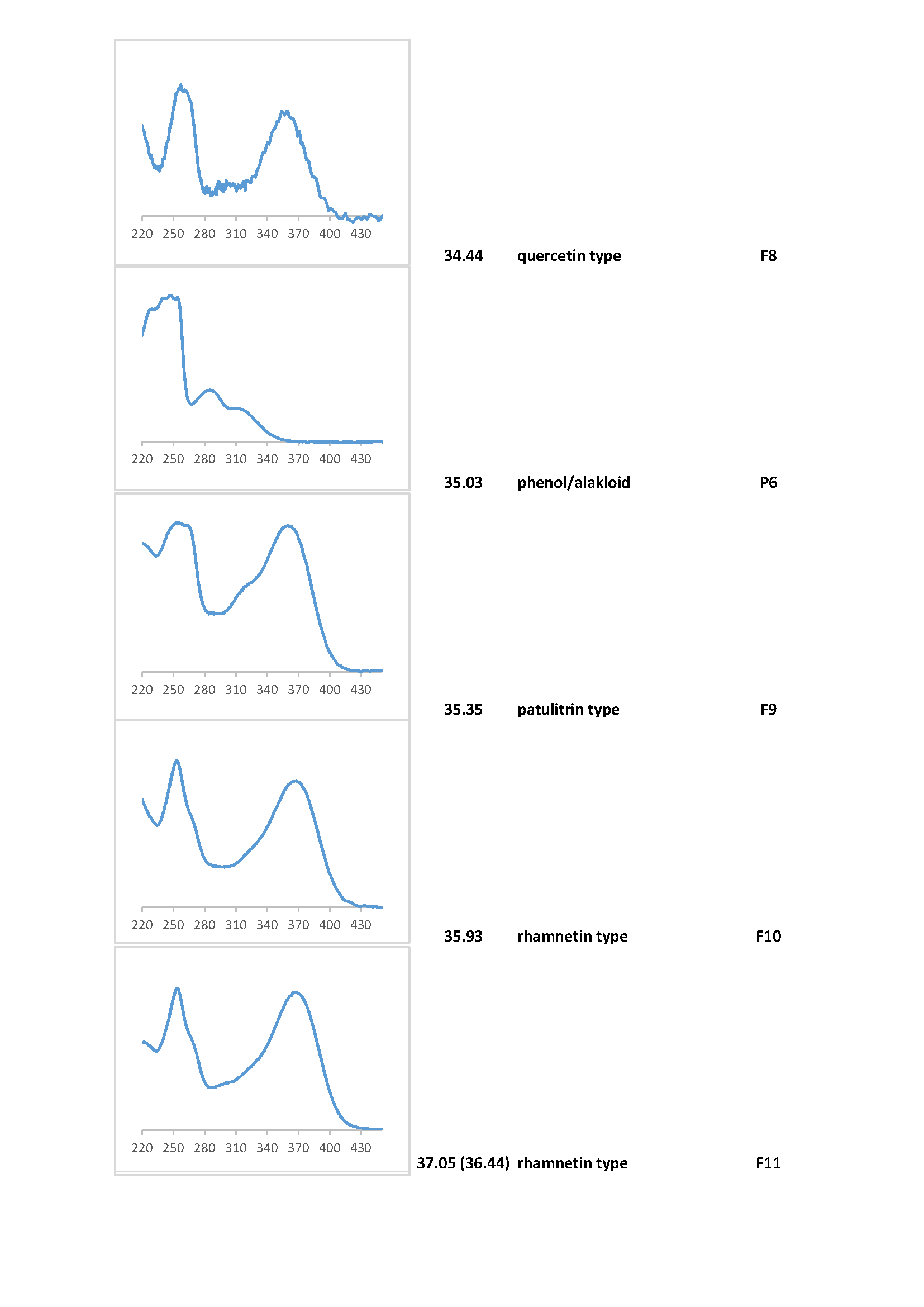
**

**
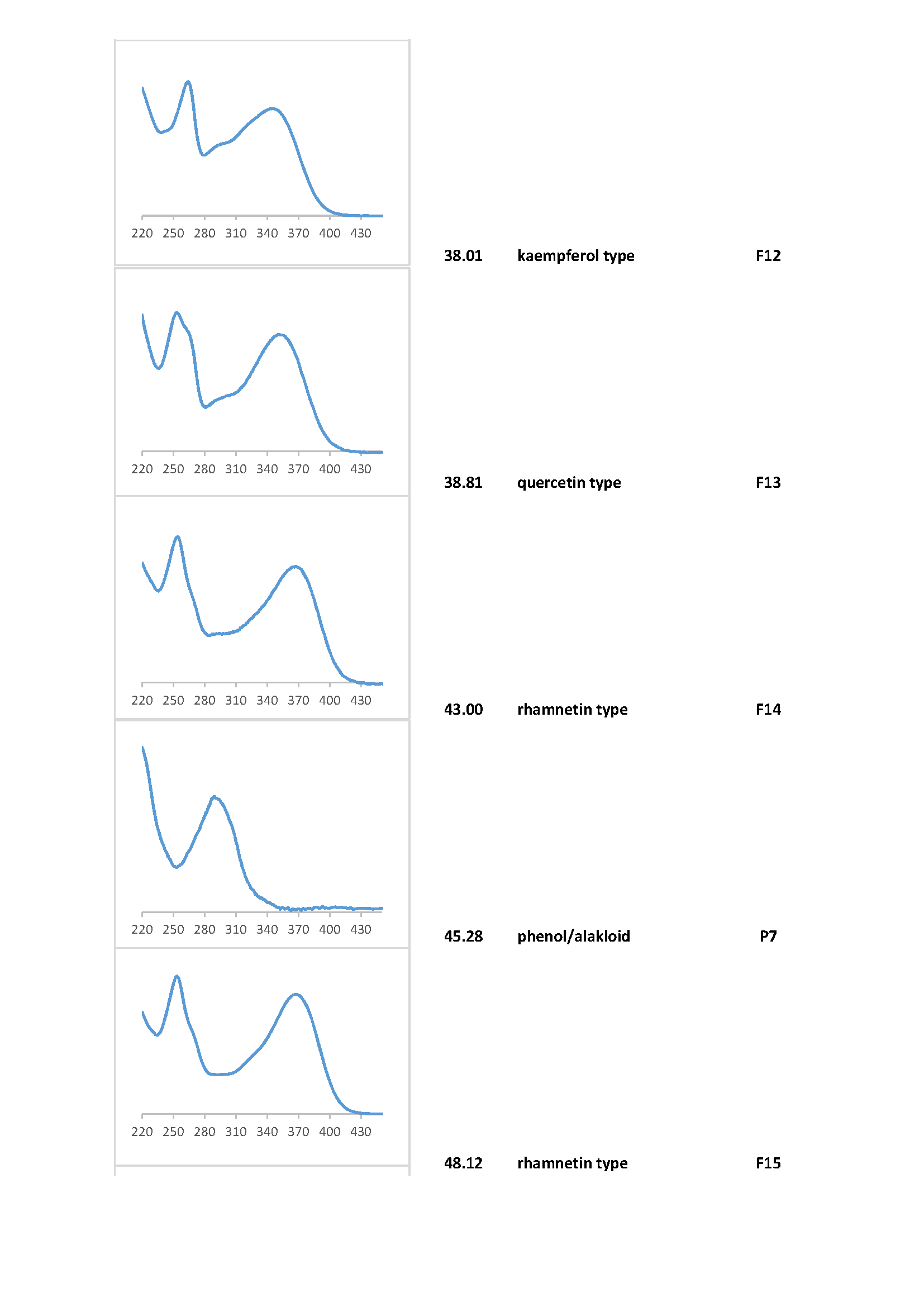

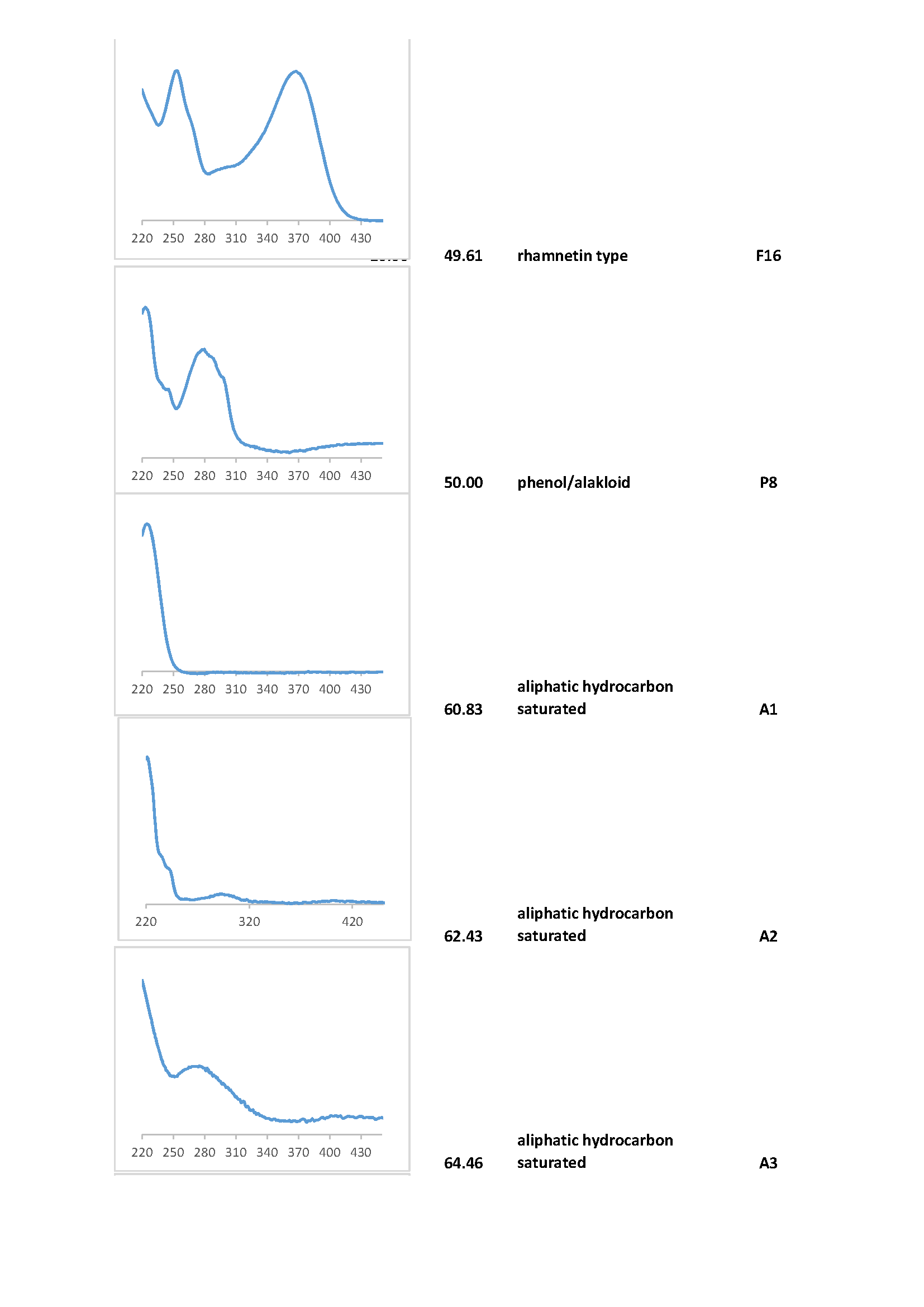
**

**
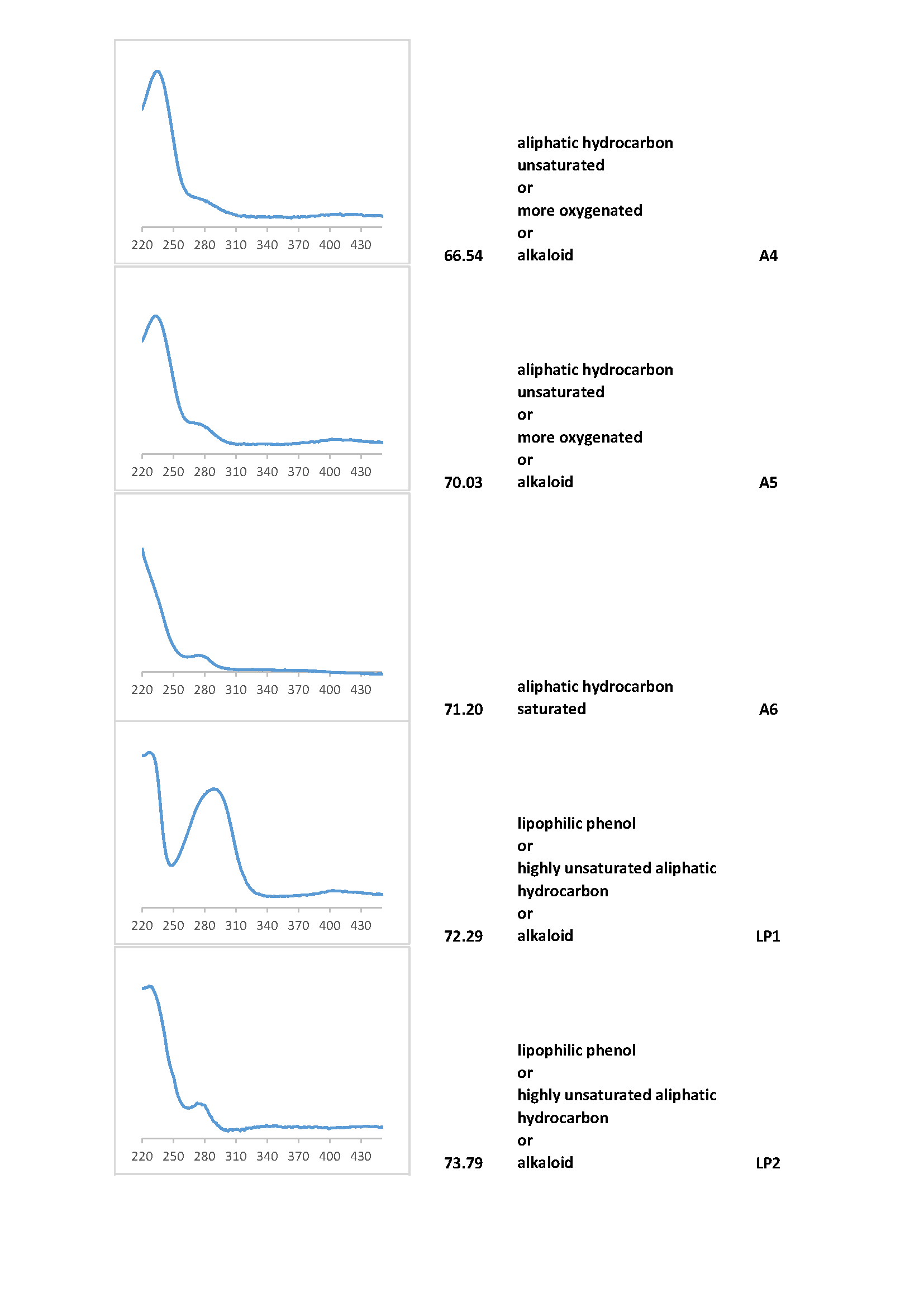
**

**
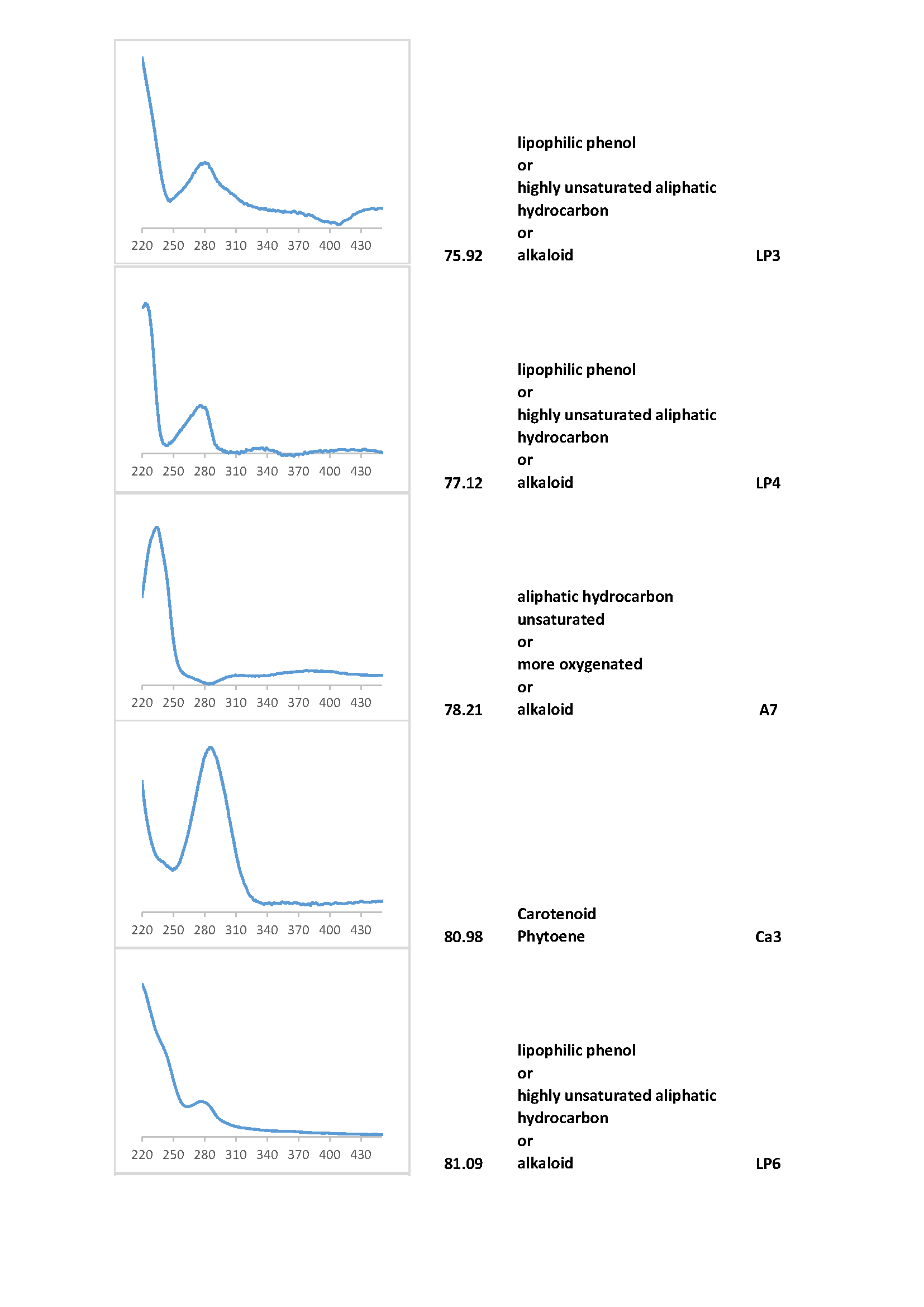
**

**
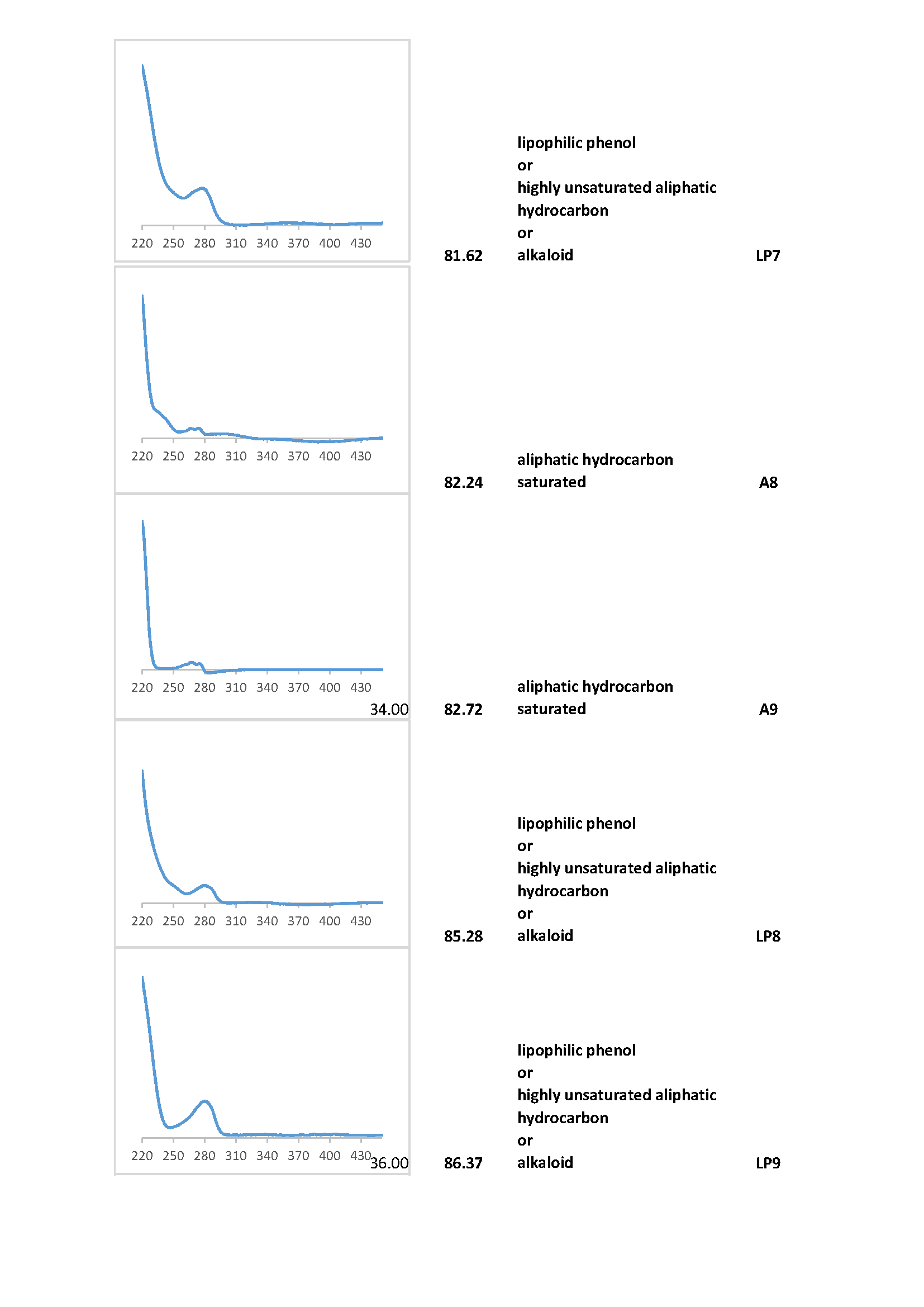
**

**
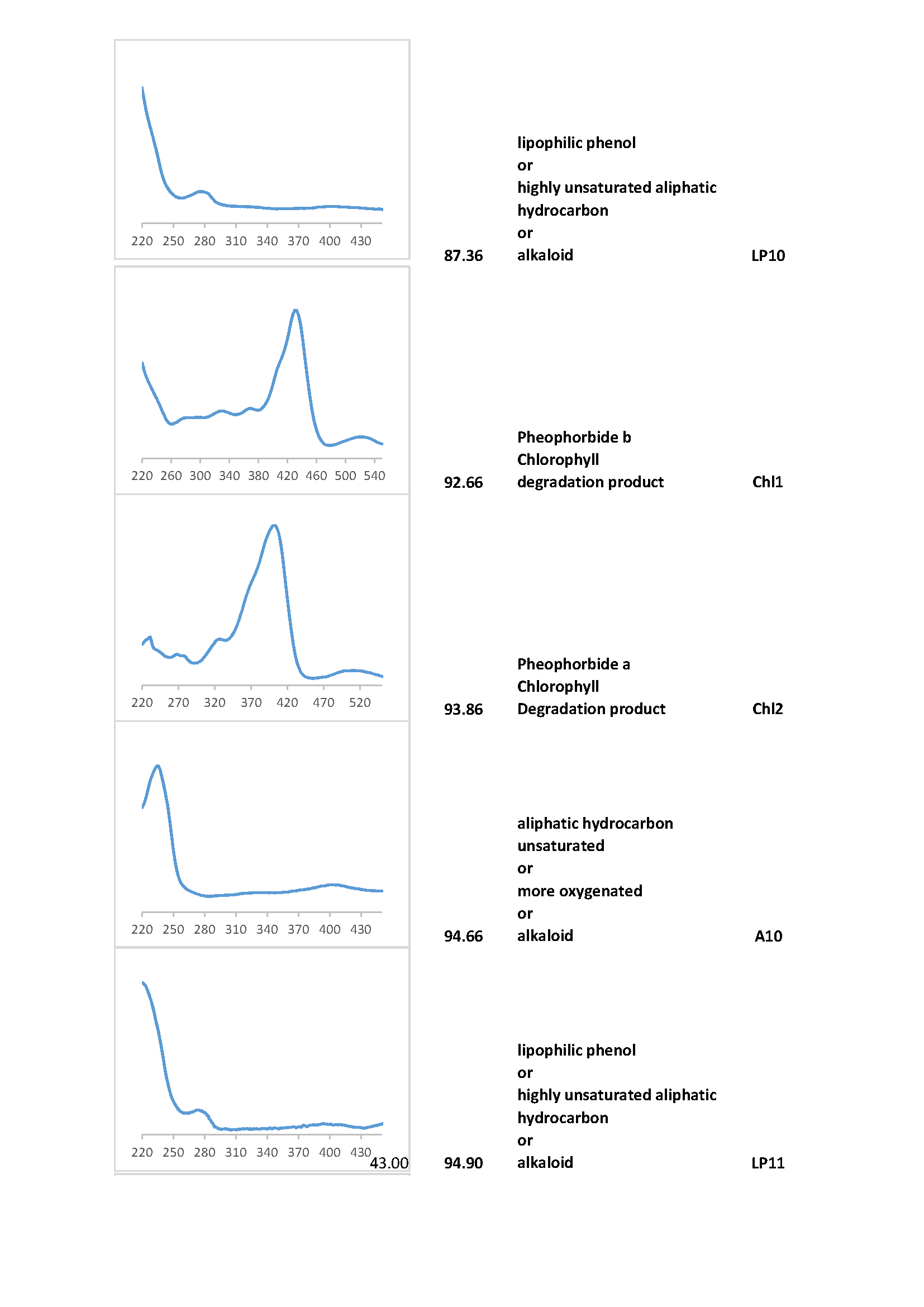
**

**
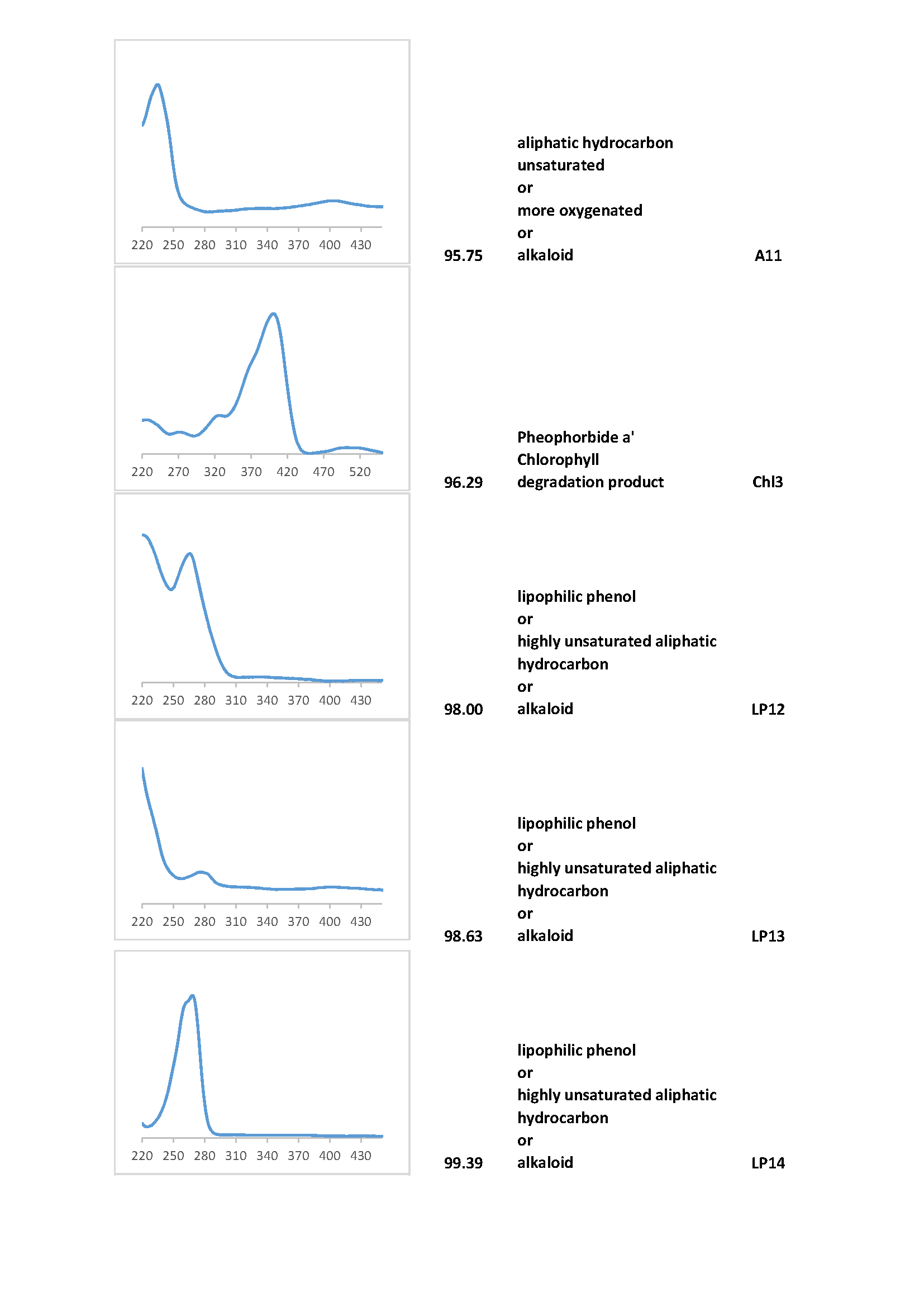
**

**
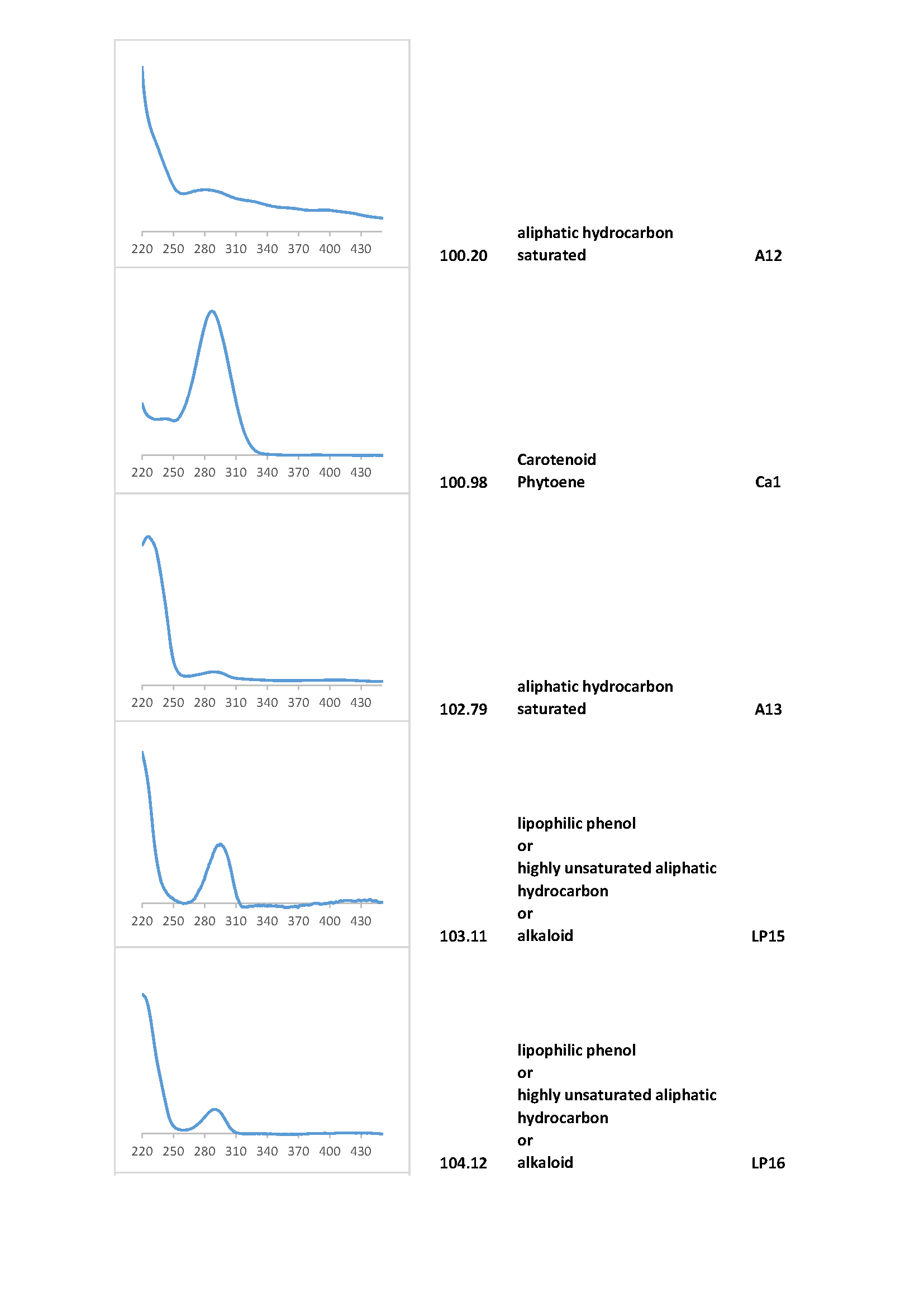
**

**
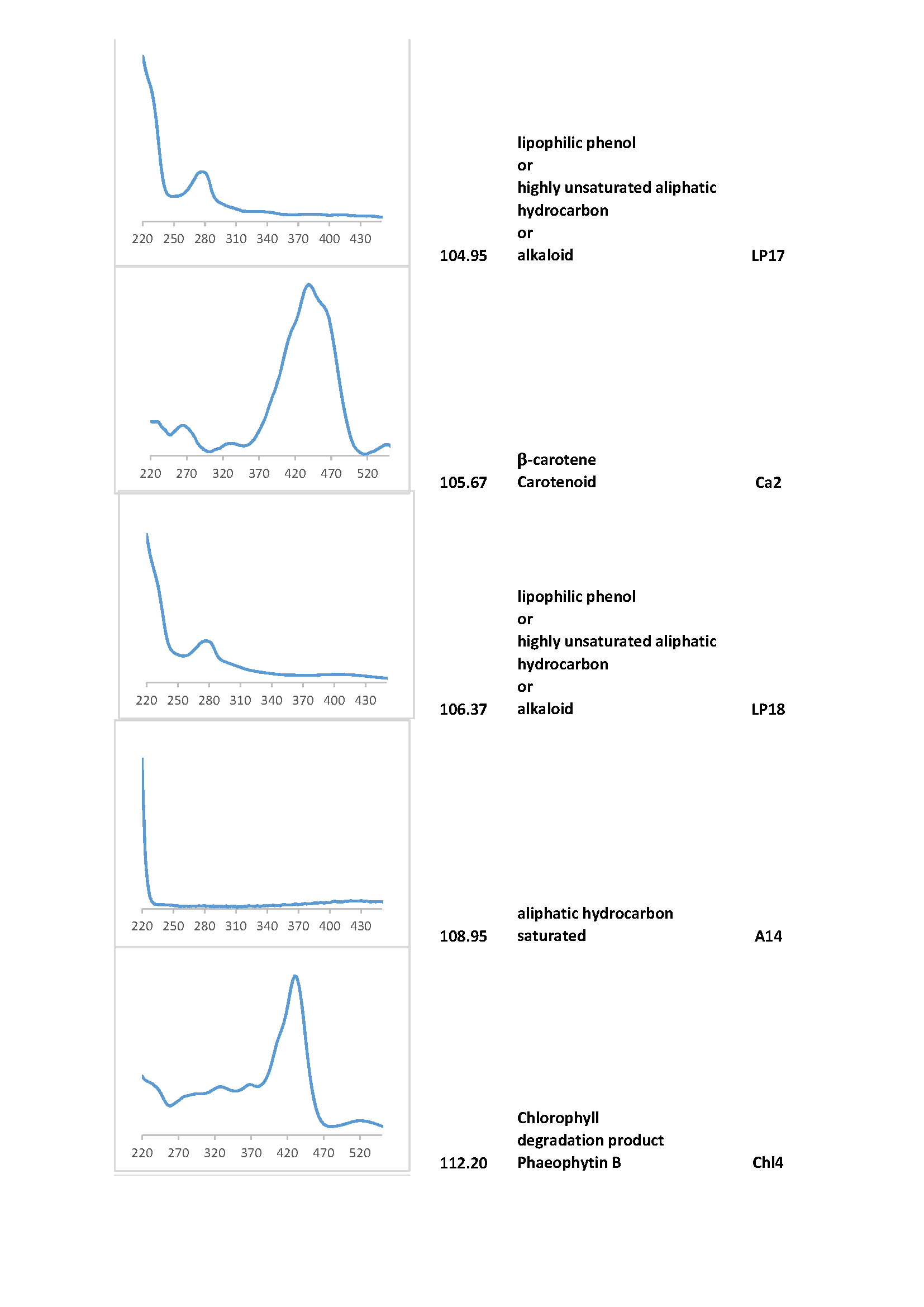
**

**
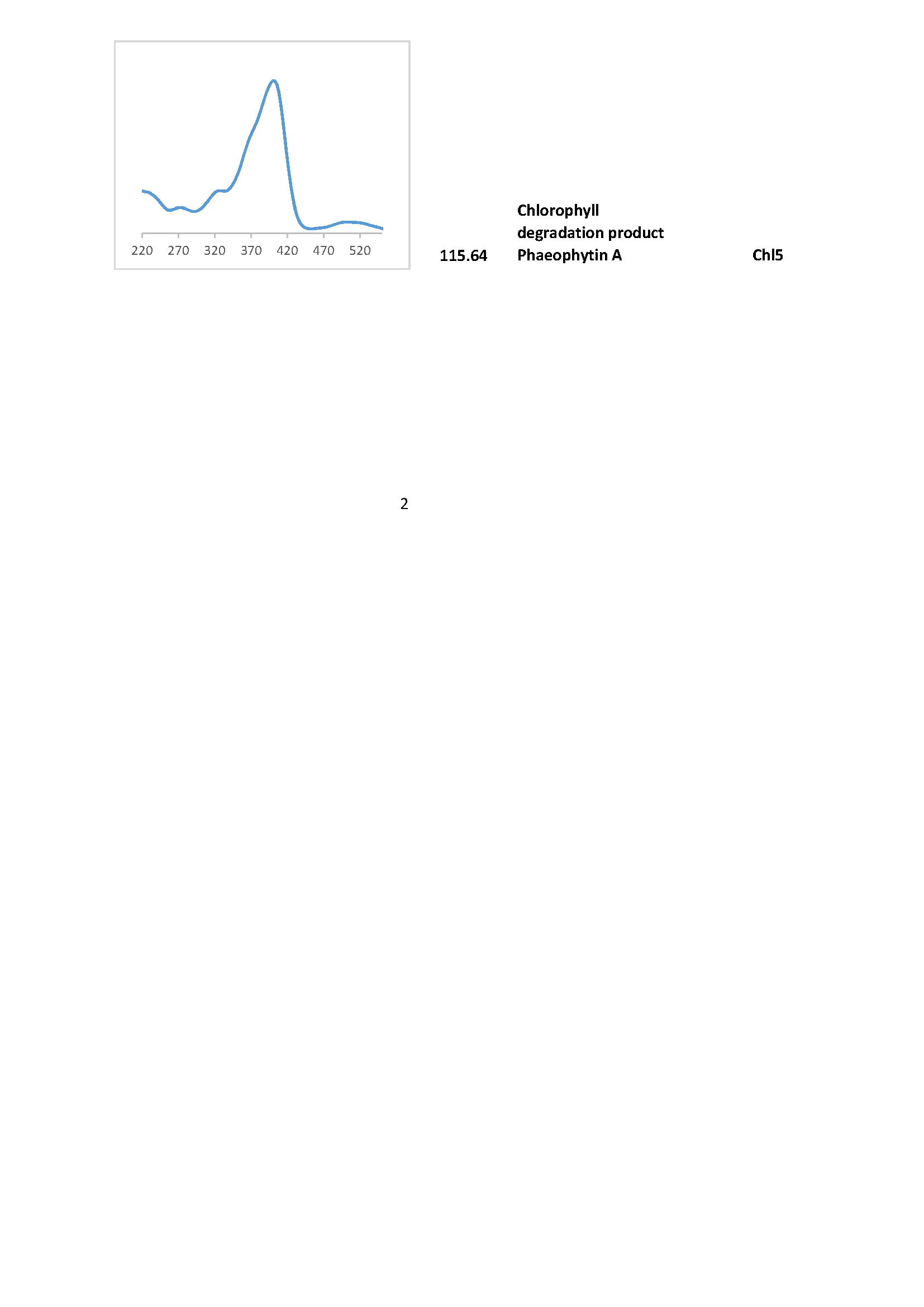
**
